# Monitoring of the field application of *Metarhizium anisopliae* in Brazil revealed high molecular diversity of *Metarhizium* spp in insects, soil and sugarcane roots

**DOI:** 10.1038/s41598-019-38594-8

**Published:** 2019-03-14

**Authors:** Natasha Sant′Anna Iwanicki, Alessandro Alves Pereira, Ana Beatriz Riguetti Zanardo Botelho, Janayne Maria Rezende, Rafael de Andrade Moral, Maria Imaculada Zucchi, Italo Delalibera Júnior

**Affiliations:** 1 Department of Entomology and Acarology, ESALQ- University of São Paulo, Av Padua Dias, 11–P.O. Box 9–13418-900, Piracicaba, SP Brazil; 2 Graduate Program in Genetics and Molecular Biology, Institute of Biology, University of Campinas, Av. Cândido Rondon 400, Cidade Universitária Zeferino Vaz 13, Campinas, SP, Brazil; 3 Department of Mathematics and Statistics, Maynooth University, Maynooth, Co. Kildare Ireland; 4 Laboratory of Conservation Genetics and Genomics, Agribusiness Technological Development of São Paulo (APTA), Rodovia SP 127 Km30, CEP: 13400-970, CP 28, Piracicaba, SP Brazil

## Abstract

The use of *Metarhizium* against sugarcane spittlebugs in Brazil is one of the most successful and long lasting biological control programs using entomopathogenic fungus in the world. However, studies to monitor the fate of this fungus on the sugarcane agroecosystem are rare, especially with respect to its persistence, efficacy in pest control and impact on the local populations of *Metarhizium*. The present study aimed at documenting the efficacy and persistence of *M. anisopliae* strain ESALQ1604 in a sugarcane field by using microsatellite molecular markers. The species diversity of *Metarhizium* was characterized in insects, soil and sugarcane roots in a sprayed and an unsprayed plot. Although the infection rates were not very high (≤ 50%), the applied strain was recovered from spittlebugs after 7, 30 and 60 days’ post-application, but accounted for only 50%, 50% and 70.5% of all insects killed by *M. anisopliae*, respectively. All haplotypes from spittlebug were associated with a single subclade of *M. anisopliae*. The highest haplotype diversity was found in soil (h = 0.989) and in the smallest in spittlebug (h = 0.779). *Metarhizium robertsii, M. anisopliae, M. brunneum*; one taxonomically unassigned lineage was found in soil and only *M. brunneum* and *M. anisopliae* were isolated from roots. This study revealed the great diversity of *Metarhizium* spp. in the sugarcane agroecosystem and the importance of the local population of *M. anisopliae* on spittlebugs management.

## Introduction

Sugarcane (*Saccharum* spp.) is one of the key crops for the Brazilian economy. Indeed, the sugar and sugarcane-ethanol industry represent 12% of the country’s Gross domestic product (GDP) and employs 4.5 million workers annually^1^. During the 2017–2018 crop season, 646.4 million tons of sugarcane are expected to be harvested from approximately 8.7 million hectares^2^. Due to the increasing number of flex-fuel vehicles and the requirement for renewable energy, the demand for ethanol predicted for 2021, according to government sources, is expected to reach 68.3 billion liters^3^ representing 2.2 times the quantity produced in 2015 (30 billion liters)^4^. In addition, there is a significant number of companies expanding business opportunities in new biorefineries, investing in R & D of bioplastics^5^ and developing studies on bioelectricity. In this promising scenario for the sector, the government has introduced incentives and programs to increase the efficiency in sugarcane production.

Among the major pests of sugarcane are the spittlebugs, especially the species *Mahanarva fimbriolata* (Stål, 1855) and *Mahanarva posticata* (Stål, 1854) (Hemiptera: Cercopidae). *M. fimbriolata* can cause losses of up to 40% in production^6^ at various stages of development during the rainy season (November to March) in Southeast of Brazil. Nymphs extract water and nutrients from the roots, causing stress that predisposes plants to steam-cracking and deterioration, while adults feed and inject toxins into leaf which cause necrosis of the leaves^7^. Furthermore, these damages change the physical quality of sugarcane used as raw material in the industry, by reducing the sugar content in stems and increasing the fiber content^8^.

The genus *Metarhizium* is an important group of entomopathogenic fungi with a worldwide distribution^9^. Products formulated with this fungus are used in agriculture worldwide, acting as biological control agents for many orders of insect pests^10^. In Brazil, *M. anisopliae* is the fungal species with the highest number of product registrations^11^, as well as being the entomopathogen most widely commercialized by companies and produced on a large scale by sugarcane mills for controlling spittlebugs (adults and nymphs). Nowadays it is estimated that 2 million hectares are treated annually with *Metarhizium anisopliae* to control spittlebugs^12^. This is considered one of the most successful biological control programs in the world.

Previous studies showed spittlebugs infection rates after the application of *M. anisopliae* strains to range from 16 to 88%^13–17^. These different results could be explained by the high variation of experimental conditions and different approaches used for monitoring and measuring fungal infection in spittlebugs. For a correct measurement of fungal infection, one needs to consider the natural infection of spittlebugs by native *Metarhizium* spp. in sugarcane fields. Therefore, without using molecular tools, it is not possible to distinguish whether spittlebug mortality is caused by the applied *Metarhizium* strain or by those already present in the sugarcane field. None of the studies mentioned above have used molecular tools to address spittlebug infections.

Molecular markers are tools able to detect DNA polymorphisms in different populations of a species. Indeed, number of investigators have already shown that it is possible to distinguish between various *Metarhizium* spp. isolates recovered from the environment using microsatellites markers^18–20^.

In Brazil, the species: *M. anisopliae*, *M. acridum*, *M. majus*, *M. flavoviride*, *M. brunneum*, *M. pingshaense, M. robertsii, M. lepidiotae, M. pemphigi*, *M. blattodeae, M. rileyi, M. alvesii* and *M. braziliense* have been identified, and three taxonomically unassigned lineages have also been found^21–28^. In sugarcane, four species have been identified so far in Brazilian soils: *M. robertsii, M. anisopliae, Metarhizium* sp. indet. 1 and *Metarhizium* sp. indet. 2^27^. Unlike the high species diversity in soil, only one clade of *M. anisopliae* (Mani 2) was observed to naturally infect spittlebugs collected from Brazilian sugarcane agroecossystem^27^. However, there are few studies focusing on *Metarhizium* ecology and molecular diversity, involving different sampling resources in sugarcane^29^. The impacts of natives *Metarhizium* in spittlebugs and the effectiveness and persistence of the applied strain of *Metarhizium* in sugarcane agroecosystem is still unknown.

The fungal genus *Metarhizium* is both entomopathogen and endophyte; they are found in soil, insects (specially infecting spittlebugs)^27^ and they are able to colonize roots of a variety of plants^30,31^, resulting in increased plant growth and providing increased tolerance against pests and diseases^30,31^. The diversity of *Metarhizium* from insects and from soils has been explored elsewhere but few studies focused on the diversity of root competent *Metarhizium*. Here, we hypothesized that there is a niche specialization in *Metarhizium*, with some species/strains more associated with entomopathogenicity above ground and other species/strains specialized on plant root association.

By monitoring the prevalence and the fate of a strain applied to the sugarcane, we also characterized the genetic variability of native populations of *Metarhizium* responsible for killing spittlebugs as well as from soil and sugarcane roots. This information will undoubtedly be useful to improve the microbial control of spittlebugs. In order to maximize the efficacy of the applied biological control strategy (inundative application of a virulent strain) or to promote natural biological pest control (development of conservation biological control strategies to favor local strains), the first step is to get a clearer picture of the diversity and dynamics of *Metarhizium* species in agricultural fields^31–35^.

In the present work our aims were therefore: (1) to determine the prevalence in soil, roots and spittlebugs populations of a commercial strain of *M. anisopliae* (ESALQ1604) sprayed on sugarcane field by using ten microsatellites as molecular markers. (2) to characterize microsatellite and species diversity of *Metarhizium* spp. in spittlebugs populations, soil and sugarcane roots in a sprayed and an unsprayed plot.

## Results

### Persistence of the applied strain in sugarcane field over a period of ninety days

**Figure 1:**
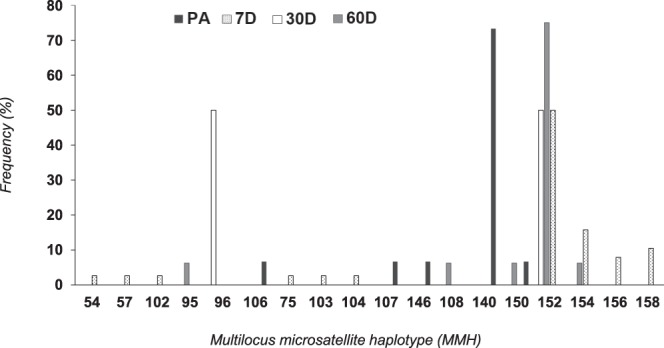
Frequency of the multilocus microsatellite haplotypes (MMH) recovered from infected spittlebugs collected on three dates before fungus application (PA) and seven (7D), 30 (30D) and 60 (60D) days after fungus application. The MMH, 152 represents the applied strain (ESALQ1604).

**Figure 2:**
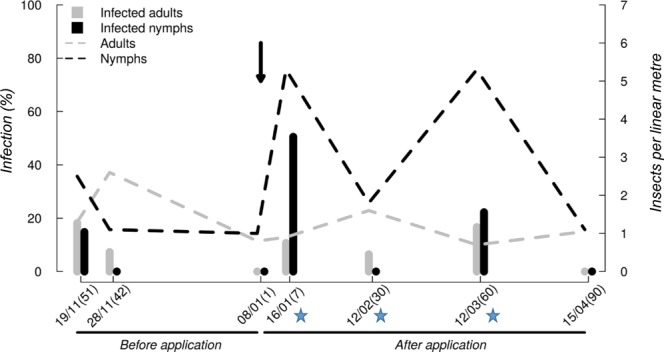
Percentage of insect infection (left y-axis) in sugarcane fields on three dates before and four dates after ESALQ1604 application, and the number of insects (right y axis) recorded by sample spot (linear meter). Percentage of fungus infection in adults and nymphs (bars) and the average of insects collected by sample point per date (dashed rows). The x-axis shows the sampling date and the days before or after the application of the fungus (Date (days before or after application)). Blue stars represent the dates in which ESALQ1604 was recovered from spittlebugs. The arrow represents the moment of application of the fungus ESALQ1604.

The applied strain ESALQ1604, represented by the multilocus microsatellite haplotype (MMH) 152 (Fig. 1), was recovered only from insects collected in the sprayed plot on all post-application dates except on day 90 (last collection) (Fig. 2). This strain represented 50% (n = 19), 50% (n = 1) and 70.5% (n = 12) of all isolates recovered from insects collected 7, 30 and 60 days after ESALQ1604 application, respectively (Fig. 1). The remaining MMH found in insects after fungus application were represented by: nine MMH (54, 57, 75, 102, 103, 104, 154, 156 and 158) recovered after seven days of application; one MMH (96) recovered after 30 days and 4 MMH (150, 154, 95 and 108) recovered after 60 days of application. Conversely, we found five MMH that represented all isolates recovered from insects on sampling dates before application (106, 107, 140, 146 and 150) (Fig. 1). Only MMH 150 was recorded from insects before and after fungal application.

### Microsatellite diversity of *Metarhizium* recovered from soil, roots and insects

As a first step, we accessed the microsatellite diversity of *Metarhizium* isolates recovered from soil, roots and insects from the sprayed and the unsprayed sugarcane plot. We genotyped a total of 308 *Metarhizium* isolates recovered from soil and roots in both plots and from spittlebugs in the sprayed plot. We did not find any spittlebugs in the unsprayed plot at sampling dates (see Supplementary Table S2).

In total, we identified 152 MMH, of which 130 were found in soil, 17 in roots and 18 in insects (Table 1). Of this total, only 13 MMH were found in isolates recovered from more than one sample group (soil, roots, insects). One MMH (140) was found in isolates recovered from insects, roots and soil, another MMH (146) was found in isolates from insects and soil, while ten MMH (134, 136, 138, 149, 162, 164, 168, 171, 172, 173) were found in isolates from soil and roots. The number of isolates recovered from soil and root from the sprayed and unsprayed plots by sampling date can be access in Supplementary Table S4. The highest MMH diversity was found in soil (h = 0.985) and the lowest in insects (h = 0.755) (Table 1). Furthermore, the analysis of molecular variance (AMOVA) showed that the genetic divergence between isolates from the different groups (soil, roots, insects) was high and significant (ФST = 0.303, p < 0.001). Additionally, the analysis of similarity (ANOSIM) reveled a significant effect of origin (insect, root, and soil) (R = 0.4263, P < 0.01), and hence there is evidence that the different origins generate statistically different assemblages of haplotypes. However, a greater proportion of the variation was observed within (69.6%) than between (30.3%) isolates from each of these groups.

**Table 1:** Estimates of haplotype diversity for *Metarhizium* spp. isolated from spittlebugs, roots and soil. N = number of isolates; NH = Number of haplotypes; NeH = effective number of haplotypes; h = haplotype diversity.

Population	N	NH	NeH	h
Insects	71	18	4.082	0.755
Root	22	17	12.737	0.921
Soil	213	130	64.720	0.985

We found only 13 MMH that occurred in soil and roots isolates from both plots (133, 137, 139, 140, 144, 146, 149, 155, 161, 162, 163, 164, 165). However, we found a high number of MMH exclusive for each plot: 46 MMH were found only in the sprayed plot. Of these, 35 occurred only in soil, five only in roots and six in roots and soil. Conversely, 75 MMH were found only in the unsprayed plot. Of these, 73 occurred only in soil, one in roots and one in roots and soil. It is noteworthy that the ratio between new MMH per isolate recovered from soil was similar between plots; sprayed plot (1:1.40), unsprayed plot (1:1.79). This can also indicates a lack of saturation of the soil sampling, suggesting that more isolates need to be characterized to fully reveal the diversity in the plots. These results indicate the existence of a high natural diversity of *Metarhizium* isolates in soil regardless of the management in sugarcane field. These findings are supported by AMOVA when considering the different plots. Although, AMOVA showed significant divergence between plots (ФST = 0.219, p < 0.001), most of the genetic variation was found within plots (78.2%) and indeed, the analysis of similarity (ANOSIM) did not reveal a significant effect of plot (R = −0.0078, P = 0.4831).

**Figure 3:**
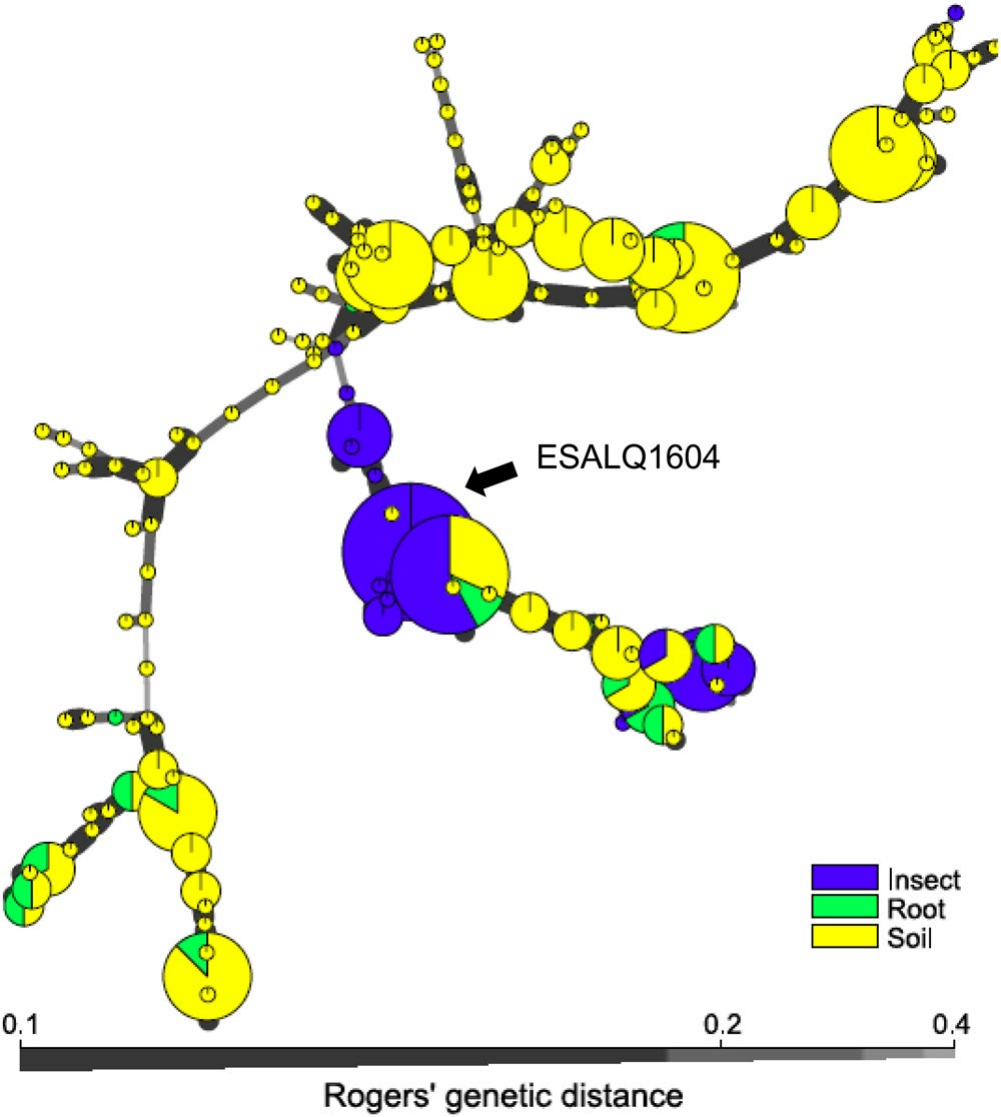
Minimum spanning network of 152 multilocus microsatellite haplotypes (MMH) of *Metarhizium* spp. Circle sizes are proportional to the number of isolates recovered for each MMH. The black arrow indicates the MMH that represent the applied strain (ESALQ1604). The width of lines between circles indicates Rogers’ genetic distance^70^. Different colors indicate the origin of isolates (insect, soil or root).

We then studied the relationships between MMH from each sample group considering all isolates recovered from the sprayed and unsprayed plots. The minimum spanning network showed the presence of three major clusters of MMH (Fig. 3). The first cluster (branch at the top of the network) consisted mostly of isolates from soil. The second cluster (the central branch) is formed from most of the MMH representing isolates from insects, but also contained MMH from soil and root isolates. The third cluster (lower branch) consisted of MMH shared by roots and soil isolates. The MMH (130) that corresponded to the isolate applied by the sugarcane mill (ESALQ 5310) in previous years on plot 1 was not found in any of the sample groups. On the other hand, we found 32 isolates from insects that shared the same MMH as that of the applied strain ESALQ1604 (152) on dates after application, thus indicating that they are the ESALQ1604. Moreover, these results showed that the 152 was found exclusively in insects. The individual minimum spanning network for each plot is presented as supplementary material (see Supplementary Fig. S3).

### Phylogeny of *Metarhizium* ssp. in the sugarcane agroecosystem

**Figure 4:**
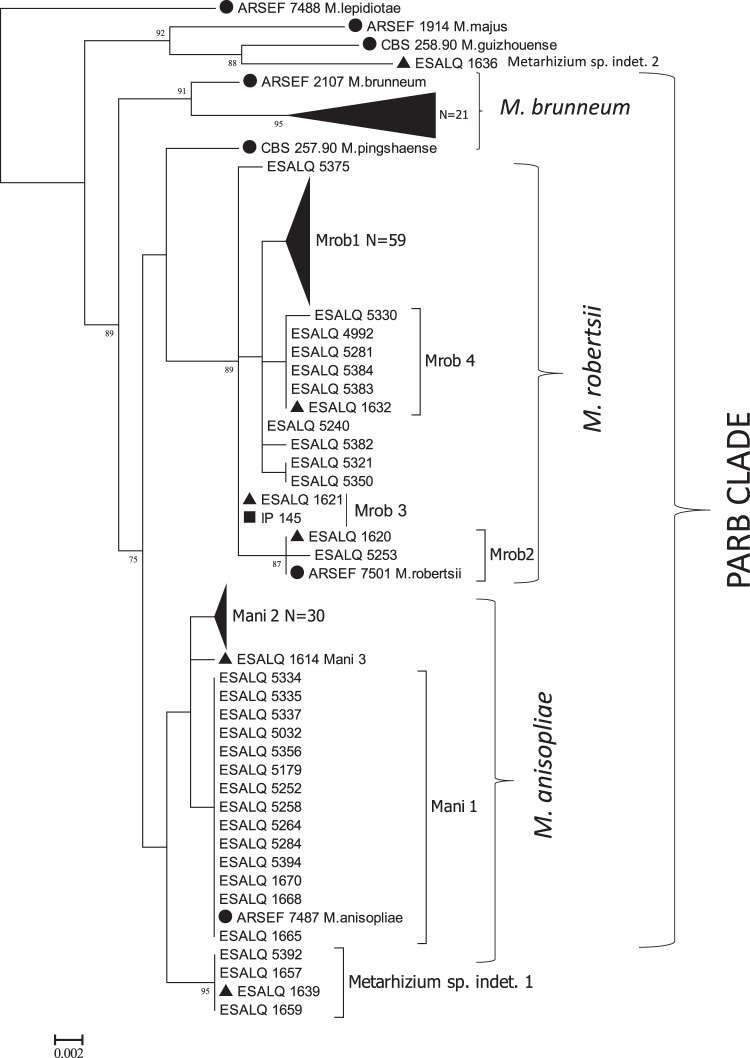
Maximum likelihood phylogeny of 5′-TEF of 128 multilocus microsatellite genotypes found in this study. A total of 142 Brazilian *Metarhizium* isolates from the ESALQ culture collection are represented. Black circles represent strains from CBS Culture Collections (n = 2) and from taxonomically validated reference accessioned in ARS Entomopathogenic Fungal Culture Collection (ARSEF, n = 7, two of them representing the clades Mani 2 (ARSEF_6347), Mrob 1 (ARSEF_727)). The black triangle represents strains previous published in Rezende *et al*.^27^ (n = 6) and black square represent a strain (IP 145) previous published in Rocha *et al*.^21^. Above branches, bootstrap support is shown for ML (>70).

We investigated the phylogenetic relationships between 128 of the 152 MMH found in *Metarhizium* isolates from soil, roots and insects, using the 5′-TEF gene (see Supplementary Table S3). The final sequence size obtained was 580 base pairs. According to the reconstruction of the maximum likelihood tree, we found three known species: *M. robertsii*, *M. anisopliae* and *M. brunneum*, plus one taxonomically unassigned lineage, but with identification in progress (*Metarhizium* sp. indet. 1) (ESALQ5392, ESALQ1657, ESALQ1659) (Fig. 4).

**Figure 5:**
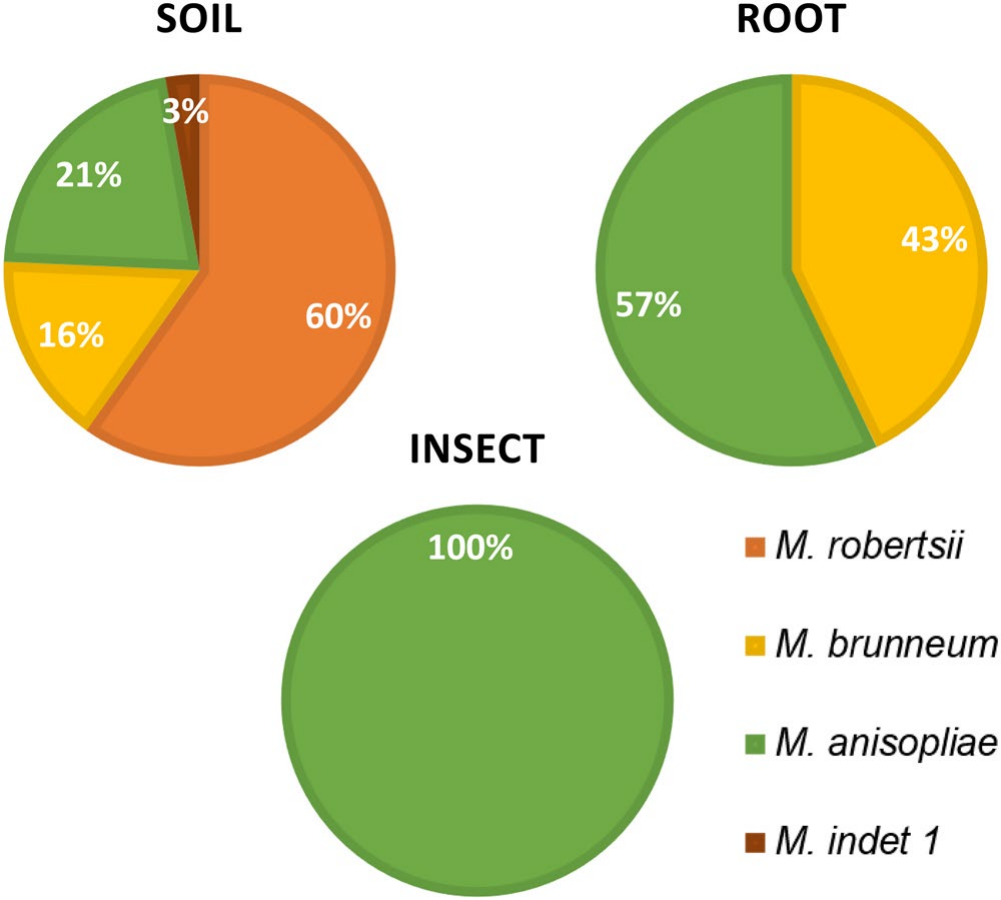
Percentage of *Metarhizium* species addressed to the haplotypes representatives isolates from soil (n = 113), roots (n = 6) and insects (n = 14) from sugarcane field.

In the soil, we found the following species: *M. robertsii* (n = 64, 60%) [distributed across the clades Mrob 1 (n = 53), Mrob 2 (n = 1) and Mrob 4 (n = 5), plus five isolates (ESALQ5375, ESALQ5240, ESALQ5382, ESALQ5350, ESALQ5321) with unresolved placement], *M. anisopliae* (n = 23, 21%) [distributed across clades Mani 1 (n = 14) and Mani 2 (n = 9)], *M. brunneum* (n = 17, 16%) and *Metarhizium* sp. indet 1 (n = 3, 3%) (Figs 2 and 3). In isolates from roots, we found *M. brunneum* (n = 4, 43%) and *M. anisopliae* clade Mani 2 (n = 3, 57%). In isolates from insects, we found only the species *M. anisopliae* clade Mani 2 (n = 14) (Figs 4 and 5).

The strain applied in previous years (ESALQ5310) and the strain applied by us (ESALQ1604) are members of clade Mani 2. In this study, we also noted in sugarcane fields the occurrence of *M. robertsii* (Mrob 1) in a beetle from Scarabaeidae family (ESALQ5168), not included in the maximum likelihood tree.

### Recombination

**Figure 6:**
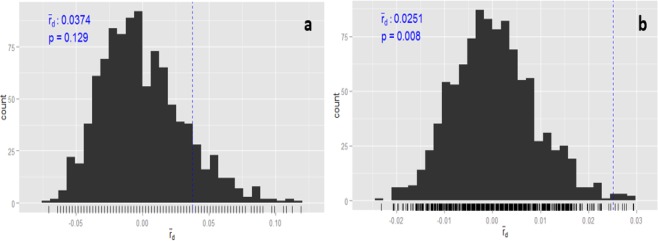
Tests of recombination for *M. anisopliae* (Mani 2), n = 14 (**a**) and *M. robertsii* (M rob 1) n = 56 (**b**) multilocus genotypes represented by isolates exclusively from soil and insect.

We also investigated whether there was any recombination among isolates from soil belonging to the main clades found in this study, Mrob1 (*M. robertsii*) and if the same occurs among isolates from insects of the clade Mani 2 (*M. anisopliae*). Significant evidence of recombination was found in isolates from insects of the clade Mani2 (IA = 0.224, p-value = 0.129; rd = 0.037, p-value = 0.129) (Fig. 6a). On the other hand, no evidence of recombination based on the IA (0.186, p-value = 0.008) and rd (0.025, p-value = 0.008) was found in Mrob1 (Fig. 6b) suggesting clonal reproduction among isolates from the Mrob1 clade.

## Discussion

In the present study, we have shown that strain ESALQ1604 of *M. anisopliae* can persist infecting spittlebugs on sugarcane for up to sixty days after the application in the field. The accuracy of these results can be attributed to the highly polymorphic microsatellite markers that we used. While this is the first time that this has been shown for sugarcane in Brazil, similar studies reporting the persistence of *Metarhizium* sp. applied to field using microsatellite markers have been reported for different crops, such as strawberries^36^ and maize^37^.

It should be noted that, in addition to 50% of spittlebugs infected by ESALQ1604, another 50% of strains recovered from spittlebugs represent native isolates of *M. anisopliae*. Indeed, we reported that 17 MMH from genotyping data of *M. anisopliae* were responsible for 20–50% of naturally infected spittlebugs; this underlines the important role of native isolates in the natural biological control of spittlebug populations. The relative importance of the native isolates in killing spittlebugs is currently under investigation in our laboratory. Taken together these findings constitute a solid basis for the development of new approaches for improving the management of spittlebugs control in sugarcane, such as applying a pool of isolates, made up of commercial and native strains, possibly resulting in higher control rates.

After 90 days of application (April 15^th^, 2015) we did not find any spittlebugs on the field; this is probably due to both the end of the rainy season in the state of São Paulo and to the lower temperatures that induce diapause of spittlebug eggs, thus decreasing the spittlebug populations in the field.

While ESALQ1604 was prevalent in spittlebugs, we did not find any this MMH in soil or in roots. The same was true for ESALQ 5310, the isolate applied by the sugarcane mill in the sprayed plot up to October 2013, 15 months before the ESALQ1604 application. Native *Metarhizium* sp. isolates can reach concentrations as high as 10^4^–10^6^ fungal propagules per gram of soil^38^. The low relative concentration and the low volume (150 L/ha) of the applied strain in relation to native isolates could be the reason why we did not find the applied strain in the soil and in roots using the present methodology. Another hypothesis to explain why we did not find the applied strain of *M. anisopliae* in the soil and in roots is the short persistence of this strain in the soil. Based on previous study, our research group has hypothesized that Brazilian *M. anisopliae* isolates are better adapted to insect pathogenicity above ground, whereas *M. robertsii* s.l. has a primarily soil-based ecology^27^. All the isolates originated from mycosed insects in that study, representing nearly 2/3 of Brazilian land area, belong to a unique clade, the *M. anisopliae* Mani2, which includes isolate ESALQ 1604 used in this study. The apparent distinct niche occupation by both species was also reported by other studies^24,36^ that demonstrated *M. robertsii* as the most abundant species in soils from different agroecosystem in Brazil. Its versatility and adaptation to the edaphic environments could be related to its capability to establish endophytic associations with plants^34^.

Microsatellite markers not only provided information about the persistence of applied strain ESALQ1604 discussed above, but also about the diversity of native isolates in soil roots and insects. The low diversity found in insects by microsatellites was supported by the sequencing data. All isolates recovered from insects in this study were allocated to the Mani 2 clade of *M. anisopliae*. These findings corroborate those of Rezende *et al*.^27^, who sequenced the 5′-TEF gene from 32 isolates recovered from spittlebugs in several regions of Brazil and observed that all of them were within the Mani 2 clade of *M. anisopliae*. Similarly, in Mexico, Hernandez-Domingues *et al*.^39^ found only *M. anisopliae* infecting spittlebugs from the genus *Aeneolamia* in sugarcane plantations. Conversely, the high MMH diversity found in soil in our study could be attributed to different factors such as the occurrence of a high number of *Metarhizium* species and specific clades identified in this study by 5´-TEF. In this environment, *M. robertsii* was the most abundant species, representing 60% of the isolates, with representative haplotypes in three out of four known clades of *M. roberstii* (Mrob1, Mrob2 and Mrob4). *M. anisopliae* was the second most common species found in soil (21%), with two know clades being represented (Mani1 and Mani2), followed by *M. brunneum* (16%) and *Metarhizium* sp. indet. 1 (3%) (Fig. 5). The high MMH diversity in soil is also supported by significant results from AMOVA analysis, where 69.6% of the variation could be explained by the source of recovered isolate (soil, root or insect) and ANOSIM analyses (significant effect of origin R = 0.4263, P < 0.01). The fact that we found only 13 MMH in more than one sample group (soil, insects or root) could indicate that the sampling was not saturated. The different management in each area seems to play an important role on the MMH diversity found in soil and roots in this study. We found in AMOVA that 22% of the MMH diversity was attributed to differences between plots. In spite of the fact that to confirm this statement, we should repeat the experiment in other areas with the same management type, these findings corroborate other studies that reported significant differences in the diversity of *Metarhizium* isolates depending on the management of the study area^20,40^.

Although low numbers of isolates were recovered from roots (n = 22), the MMH diversity was higher (h = 0.921) than it was for insects (h = 0.755). We found two species in roots, *M. anisopliae* Mani 2 (40%) and *M. brunneum* (60%). The occurrence of *M. anisopliae* in roots had been recorded previously in several studies^41–43^ as well as *M. brunneum*^43,44^. However, the higher relative prevalence of *M. brunneum* isolates in roots (43%) compared to soil (16%), could indicate that *M. brunneum* is more adapted to the root environment in the sugarcane agroecosystem. It is widely known that *Metarhizium* has a bifunctional lifestyle as an insect pathogen as well as a plant endophyte. Their evolutive relation with the fungal grass endosymbionts *Claviceps* and *Epichloë*^45^ and the large number of genes for plant degrading enzymes within their genomes^46^ points to insect pathogenicity as an adaptation to access a specialized source of nitrogen (i.e. insects), or other insect derived nutrients, and to effectively barter these insect-derived nutrients to access plant carbohydrates^47^. Besides the nutrient exchange, this association may bring other benefits such as a larger number of lateral roots and root hair formation, plant height, and shoot/root dry weight acting as plant growth promoters, improving the growth and productivity of plants^31,35^, and protecting them against microbial pathogens^30^ as well. So far, only one study has demonstrated the endophytic colonization of sugarcane plants using *B. bassiana* which provided an enhanced numbers of roots^48^. Therefore, observation of the endophytic association of *Metarhizium* in sugarcane may indicates the potential for future investigations of its use as a bioinoculant.

Tests of recombination were performed for the two most representative clades of *Metarhizium* sp. identified in this study. The MMH diversity suggests clonal reproduction among isolates of the clade Mrob 1 from soil, while the patterns of diversity is consistent with the occurrence of recombination among isolates of the clade Mani 2 from insects. Kepler *et al*.^20^ also found a strong clonal signature for the Clade 1 of *M. robertsii* (Mrob 1), while commenting that a predominance of clonal structure in *Metarhizium* communities is expected in most agroecosystems. According to these authors, the clonal structure is consistent with the prevailing practice of releasing single *Metarhizium* genotypes for the biological control of insects. It is noteworthy that both of the strains used for biological control of spittlebugs (ESALQ1604 and ESALQ5310) were identified as members of the Clade 2 of *M. anisopliae* (Mani 2), which showed signs of a recent history of recombination. Despite the prominence of non-recombination in the life histories of *Metarhizium* species of PARB clade^20,49^, a recent study showed the presence of mating type idiomorphs which supports the idea that all PARB species are also fundamentally outcrossing^50^, but the circumstances in which sexual reproduction may occurs remains unknown.

In summary, we have shown that the applied strain of *M. anisopliae* persists in infecting spittlebugs for up to sixty days; Native *M. anisopliae* isolates control between 20–50% of spittlebug populations and the highest diversity of *Metarhizium* is found in the soil environment. Nonetheless, for the first time *M. brunneum* was recovered from sucarcane soil in Brazil. These results constitute a major step in understanding the ecology of *Metarhizium* in sugarcane fields and the destination of *M. anisopliae* application in the environment.

## Methods

### Experimental field

The experiments were conducted in a sugarcane field of the Iracema Mill (São Martinho Group) located in Iracemápolis city in the state of São Paulo, Brazil. In the sugarcane field, two plots of 9,800 m^2^ each, 700 m apart were delimited. The first one (sprayed plot), located at the coordinates 22°36′10″S, 47°33′17″W and the second (unsprayed plot) at the coordinates 22°36′30″S, 47°33′23″W. The difference between plots consisted of spittlebug control and sugarcane harvesting method which implies burning or not burning the sugarcane before harvesting. Sprayed plot was an area annually treated with *Metarhizium anisopliae*, strain ESALQ 5310 and harvested mechanically. The unsprayed plot was located in an area where *M. anisopliae* has never been applied before and the sugarcane is harvested manually. For manual harvesting, the sugarcane crop needs to be burned prior to cutting the stems in order to eliminate the straw. The soil type of both plots is a Red Latosol and there is no irrigation whatever on these plots.

### Strain selection and application of *M. anisopliae* in the field

The *M. anisopliae* strain ESALQ1604 from the Collection of Microorganisms of the Laboratory of Pathology and Microbial Control of Insects at the Department of Entomology and Acarology of the University of São Paulo was chosen for field application. The ESALQ1604 was isolated in Alagoas, a state located in the Northeast of Brazil far from the released site. This strain is commercially available (Biotech G^®^ produced by Biotech Controle Biologico) for the control of *Mahanarva* spp. This strain proved to be a unique haplotype among 318 *Metarhizium* sequences of the nuclear intergenic MzIGS3 locus (unpublished data). We understand that due to its unique location in the MzIGS3 phylogenetic tree, this strain could be genetically different from native strains recovered from sugarcane fields and therefore easier to distinguish with microsatellite markers.

Aerial conidia of strain ESALQ1604 was produced on 25 kg of parboiled rice^10^. The application of fungus was carried out in the sprayed plot on January 9^th^, 2015 at a temperature of 28 °C. A volume of 150 L ha^−1^ of 3.72 × 10^6^ viable conidia/mL was applied using a self-propelled sprayer tank. To provide a better fungus-application coverage, the self-propelled bar was placed two meters above the sugarcane leaf height.

### Insect, soil and roots sampling

#### Soil and roots sampling

In order to characterize native *Metarhizium* spp. community in both different managed plots, samples of sugarcane soil and roots were collected at four different dates. The first sample was collected on December 18^th^, 2012. The second sample was collected on October 9^th^, 2014, 90 days before applying the strain ESALQ1604 in the sprayed plot. The third sample was collected on February 9^th^, 2015, 30 days after fungus application. The final sample was collected on April 10^th^, 2015, 90 days after fungus application.

The methodology for soil and root sampling was proposed by Jürg Enkeli (Agroscope, Institut für Nachhaltigkeitswissenschaften INH, Zurich, Switzerland) for the project on global phylogeography of *Metarhizium*. Briefly, within each plot we delimitated an area of 3,000 m^2^ wherein points were sampled every five meters in four 100 m rows (see Supplementary Fig. S1). Rows were at a distance of 10 m from each other, resulting in 20 sampling point per row, totaling 80 sampling per plot at the dates before fungus application. After fungus application, soil samples were collected at every ten meters in each row, instead of five meters, resulting in 40 samples per plot and date. Twenty samples of roots were collected in each plot at dates before fungus application and 30 and 90 days after fungus application. Roots were not obtained in the unsprayed plot after 90 days because all the plants were removed from that field as they achieved the maturation point for being harvested.

Samples were individually packed in plastic bags and stored in a cooler until transported to the laboratory, where they were kept in a refrigerated room until processing.

#### Insects sampling

Adults and nymphs of *M. fimbriolata* spittlebugs were collected in the sprayed plot on seven dates, three before and four after fungus application. The first three collections aimed to evaluate the rate of spittlebugs natural infection by *Metarhizium* sp. and were conducted 51, 42 and one day before the fungus ESALQ1604 application. The last four collections were conducted after 7, 30, 60 and 90 days of fungus application. We didn´t find spittlebugs in the unsprayed plot on any sampling date.

Spittlebugs’ sampling methodology followed the scheme used to monitor insect population adopted by the sugarcane mill (see Supplementary Fig. S2). On average, 23 sample points representing one linear meter in each row, and spaced 16 meters from each other, were evaluated for each sample date in the sprayed plot. In each sample point, adult and nymph spittlebugs were collected and recorded.

Nymphs were collected with forceps and individually placed into containers with soil that was attached to the sugarcane roots to keep the humidity. These containers were immediately stored in Styrofoam boxes. The same procedure was performed for adult sampling, but instead of soil we used a piece of sugarcane leaf. In the laboratory, nymphs and adults were kept in quarantine on roots and leaves of sugarcane seedlings grown in a vegetable substrate until their death. Seedling replacement was done when necessary. Spittlebugs cadavers found in the sprayed plot with visible sporulating conidia of *Metarhizium*, were stored individually in labeled microtubes and kept in a refrigerator at 4 °C.

### Isolation of *Metarhizium* species from soil, roots and insects

#### Soil and roots

Insect baiting method was used to recover *Metarhizium* species from soil and root samples. For baiting we used third or fourth larvae instar of *Tenebrio molitor* (Linnaeus, 1758) (Coleoptera: Tenebrionidae), following adapted techniques from Zimmermann^51^ as well as from Meyling and Eilenberg^52^. Ten larvae of *T. molitor* were transferred to plastic pots with 140 mL capacity, containing 80 grams of soil sample or roots. Before adding to pots, roots were washed with sterile distilled water to remove any excess soil, cut into small pieces (approximately 0.5 cm). The pots were stored in climatic chamber for 14 days at 26 ± 1 °C and 12 h photoperiod. They were turned upside down daily during the first week to induce larvae movement and increase its contact chance with the fungi present in the samples. Assessments of larva mortality were done every 3 days. The soils were moistened with sterile distilled water when necessary. Dead larvae were sterilized in 5% sodium hypochlorite for 1 minute, dipped three times in sterile distilled water and then individually placed in 24-well cell culture plates for confirmation of mortality by *Metarhizium* fungus. Sporulated larva cadavers were placed in microtubes. A small portion of the conidia from cadavers were transferred to Petri dishes containing potato dextrose agar (PDA) medium to obtain pure colonies.

#### Insects

Spittlebugs adults and nymphs dead during quarantine period were superficially sterilized as described to the IB method, placed in a 24 wells cell culture plates and, incubated in a climatic chamber (26 ± 1 °C and photoperiod of 12 h) for seven days. After *Metarhizium* sporulation over cadavers, conidia were spread in PDA medium plates using platinum sterile loops until obtain pure colonies.

*Metarhizium* Isolates are cryopreserved at −80 °C in the Collection of Entomopathogenic Microorganisms “Prof. Sérgio Batista Alves”, College of Agriculture Luiz de Queiroz (ESALQ), Brazil.

#### DNA extraction

DNA was extracted from conidia obtained from pure colonies of isolates recovered by “Insect baiting” method as well as from infected spittlebugs. We selected isolates from one or two infected *T. molitor* larvae of each soil or root sample collected in plots 1 and 2. All isolates recovered from spittlebug cadavers had their DNA extracted. For molecular analysis, we also extracted DNA of 33 isolates from the first soil samples collected on December 18, 2012, in which five were recovered from the sprayed plot and 28 from the unsprayed plot.

The method described by Kepler *et al*.^23^ was used for DNA extraction, with some modifications. Conidia were inoculated on a filter paper (Grade 2 - Whatman) in small Petri dishes (3 cm in diameter) containing Difco^®^ PDA. Then, the Petri dishes were kept in climatic chamber for 7 to 10 days at 26 ± 1 °C with 12 h photoperiod. The conidia were scraped from the paper filter using a scalpel blade and then transferred to 2 mL microtubes (screw cap Sarstedt Micro Centrifuge Tube). To each microtube, 350 µL of extraction buffer were added (2.1 g of sodium metasilicate (Sigma^®^), 0.5 g of citric acid (Sigma^®^), 2.64 mL of 2-butoxy-ethanol, ethylen glycol butyl ether (Sigma^®^), 13.5 mL of 1 M Tris-HCl, pH 7.0 and 200 mL of sterile distilled water), diatomaceous earth, 1 mm and 2.3 mm zirconia-silica beads (BioSpec products). The microtubes were sealed and placed on the L-Beader equipment 24 (Loccus Biotechnology) for three cycles of ten seconds at a speed of 5.5 m/s. After homogenization and cell lysis, the tubes were immediately placed in a hot bath (100 °C) for 10 min and then centrifuged for 10 min at 14.000 g. After centrifugation, samples were immediately placed in a cold rack. An amount of 175 µL supernatant was removed from each microtube. The DNA was transferred to a new sterile microtube and stored in a −20 °C freezer for later use.

#### Genotyping

Microsatellite molecular markers were used to characterize the *Metarhizium* diversity of isolates recovered from soil roots and spittlebugs and to distinguish the applied strain, ESALQ1604 from other indigenous isolates. Initially, 23 microsatellites described by Oulevey *et al*.^53^ and Enkerli *et al*.^54^ were tested in ten isolates obtained in this study, including the ESALQ1604. Of these, we selected the ten microsatellites that had the highest peak quality for most of the isolates tested when analyzed with GeneMarker v1.95 (SoftGenetics LLC). (Table 2) We genotyped the samples by multiplexing PCR products from different microsatellite loci, which were labeled with fluorescent dyes (NED, FAM and HEX) attached to the 5′ end of the M13 universal primer sequence (5′-CACGACGTTGTAAAACGAC-3′) following Schuelke^55^.

**Table 2:** Selected microsatellites organized in three Multiplex groups. Average size of the fragments obtained by each microsatellite, assigned fluorescence, motif region and reference article.

Multiplex	Locus	Base repeat length	Fluorescence	Repeat motif	Reference
Multiplex 01	Ma2054	239	HEX	GT)_14_/(ATAC)_4_/(TATG)_6_	Oulevey *et al*.^53^
	Ma142	111	HEX	(CA)_8_(CGC)_5_	Enkerli *et al*.^54^
	Ma2097	191	FAM	(GT)_10_(T)_6_G(T)_4_	Oulevey *et al*.^53^
	Ma2099	251	NED	(GT)_4_/(GT)_4_AT(GT)_6_	Oulevey *et al*.^53^
Multiplex 02	Ma2064	163	HEX	(T)_6_A(T)_2_A(T)_9_A(T)_7_(GT)_15_	Oulevey *et al*.^53^
	Ma2065	145	FAM	(GT)_4_GA(GT)_8_GA(GT)_3_	Oulevey *et al*.^53^
	Ma165	139	NED	(CA)_4_	Enkerli *et al*.^54^
Multiplex 03	Ma2296	142	HEX	(CT)_8_CCAT(CT)_7_	Oulevey *et al*.^53^
	Ma2292	197	FAM	(T)_5_C(T)_7_/(CT)_6_C(CT)T(CT)_2_	Oulevey *et al*.^53^
	Ma2063	141	NED	(GT)_11_	Oulevey *et al*.^53^

DNA samples were diluted in water at 1:10. Each microsatellite region was amplified in a thermocycler, the MyCyclerTM Thermal Cycler mark (Bio-Rad, USA), with the following program: 1 cycle of denaturation at 95 °C for 2 min followed by 36 cycles of denaturation at 94 °C for 30 seconds, annealing at a temperature depending on the primer (ranging from 56 to 60 °C) for 30 seconds and extension at 72 °C for 40 seconds. To incorporate the fluorescently-labeled M13 primer, additional 10 cycles at 94 °C for 40 seconds, 53 °C for 40 seconds and 72 °C for 40 seconds was added to the PCR program after the initial 36 cycles. The extension cycle at 72 °C for 7 min was added at the end.

Genotyping was conducted in the Laboratory of Molecular Biology and Genetic Engineering Center (CBMEG) of the University of Campinas (UNICAMP), São Paulo (SP), Brazil and the results were analyzed with GeneMarkerV1.95 (SoftGenetics LLC).

#### Analysis of genotyping data and recombination

Genotyping data was organized on Microsoft Excel 2010, and analyzed with GenAlEx 6501^56^. The number of multilocus microsatellite haplotypes (MMH) was obtained for the groups of insects, soil and roots isolates. To determine the genetic diversity in each group, evaluations of MMH diversity were conducted and the following estimates were obtained: N = number of isolates; NH = Number of MMH; NEH = effective number of MMH and h = MMH diversity. The effective number of MMH is the expected number of MMH considering the lowest sample number (among groups), estimated on the basis of rarefaction^57^.

To illustrate the MMH diversity, a Minimum spanning network was created using the statistical analysis program R^58^, using the package poppr^59^.

Two analyses of molecular variance (AMOVA) were performed with the package poppr^59^ in R^58^. For the first AMOVA we considered two hierarchical levels (between groups and within groups) with ten microsatellite scored for each of the 306 isolates obtained of *Metarhizium* spp. recovered from the different groups: soil samples (n = 213), roots (n = 22) and insects (n = 71). For the second AMOVA, we considered two hierarchical levels (within plots and between plots) including samples from soil (n = 213) and roots (n = 22). Additionally, we performed the analyses of similarity (ANOSIM) to compare the assemblages of haplotypes between the sprayed and the unsprayed plot, and between haplotype origin (insect, root, and soil), using package vegan^60^ for R^58^.

The occurrence of recombination within *Metarhizium* isolates was evaluated using the index of association (IA)^61,62^ and rd^63^, with the package poppr^63^ for R^58^. The procedure of clone-correction was performed before the analysis, and significance was calculated based on 999 permutations.

#### Amplification, sequencing, editing and multiple alignments

To access the species diversity of *Metarhizium* spp. we sequenced the forward strand of the elongation factor 5′-TEF region. One representative isolate of each MMH were chosen randomly within all isolates that represented each MMH for sequencing. The amplification reaction was performed using the primer pair: EF1T (5′-ATGGGTAAGGARGACAAGAC-3′) and EF2T (5′-GGAAGTACCAGTGATCATGTT-3′)^64^. The PCR was conducted with a final volume of 25.0 µL, containing 14.85 µL of sterile distilled water, 5.0 µL of 1X GoTaq®ReactionBuffer, 0.5 mM of dNTP mix, 0,15 μL of GoTaq®DNA polymerase (5U/µL) (Promega), 0.75 mM of each of primer diluted at 10 pmol/µL and 3 µL of DNA diluted in a 1:5 proportion. The PCR program consisted of an initial cycle of denaturation at 94 °C for 3 min, followed by 35 cycles of denaturation at 94 °C for 40 s, annealing at 55 °C for 45 s, and polymerization at 72 °C for 45 s, ending with an extension at 72 °C for 10 min. The quality and size of the final products of the PCRs were analyzed by electrophoresis on agarose gels (1% w/v).

The PCR products were purified by an enzymatic digestion with Illustra Kit ™ ExoProStar 1-Step (GE Healthcare, São Paulo, Brazil) according to the manufacturer’s instructions and prepared for sequencing with Big Dye Terminator v3.1 Cycle Sequencing system (Applied Biosystems, Foster City, CA, USA). Sequencing was performed using the ABI3500xl Genetic Analyzer equipment (Applied Biosystems^®^ 103) from the Laboratory of Molecular Biology and Genetic Engineering Center (CBMEG) of the University of Campinas (UNICAMP), São Paulo (SP). The sequences obtained were manually edited and the multiple alignment was constructed using the ClustalW tool with BioEdit version 7.2.5^65^.

The Maximum Likelihood analysis (MV) was performed using GUI RAxML v.1.3^66^ implementing the evolutionary model GTR + G. The analysis was conducted considering “gaps” as missing data and support for the obtained relationships was accessed with 1000 replicates of the “rapid bootstrap” algorithm^67^. The generated tree was viewed and edited with MEGA6^68^.The nodes were considered to have good statistical support when the values of bootstrap were ≥70%^69^.

## Supplementary information

Supplementary material

## Data Availability

The sequences used in this study are available for download from the GenBank database of the National Center for Biotechnology Information (http://www.ncbi.nlm.nih.gov/genbank/). See Supplementary Material, Table S3 for the accession numbers of all samples included.

## References

[1] Uniao da industria de cana-de-acucar, http://www.unica.com.br/noticia/871626920312979436/liderancas-do-setor-sucroenergetico-cobram-politicas-publicas-em-audiencia-no-senado/ (2012).

[2] Conab. Acompanhamento da Safra Brasileira de Cana-de-Açúcar – Quarto Levantamento da safra, http://www.conab.gov.br/OlalaCMS/uploads/arquivos/15_04_13_08_45_51_boletim_cana_portugues_-_4o_lev_-_14-15.pdf (2015)

[3] Goldemberg J, Mello FFC, Cerri CEP, Davies CA, Cerri CC (2014). Meeting the global demand for biofuels in 2021 through sustainable land use change policy. Energy Policy.

[4] Lopes ML (2016). Ethanol production in Brazil: a bridge between science and industry. Brazilian J. Microbiol..

[5] Silva ATBD, Spers RG, Wright JTC, Costa PRD (2013). Cenários prospectivos para o comércio internacional de etanol em 2020. Rev. Adm..

[6] Dinardo-Miranda, L. L., Garcia, V. & Parazzi, V. J. Efeito de Inseticidas no Controle de *Mahanarva fimbriolata* (Stål) (Hemiptera: Cercopidae) e de Nematóides Fitoparasitos na Qualidade Tecnológica e na Produtividade da Cana-de-Açúcar. *Neotrop. Entomol*. 609–614 (2002).

[7] Alves R, Teixeira CGS (2014). Primeiro registro das espécies de cigarrinhas-da-raiz da cana-de-açúcar Mahanarva spectabilis (Distant) e *Mahanarva liturata* (Le Peletier & Serville) atacando canaviais na região de Goianésia (GO), Brasil. Agric. Entomol..

[8] Dinardo-Miranda LL (2014). Resistance of Sugarcane Cultivars to *Mahanarva fimbriolata* (Stal) (Hemiptera: Cercopidae). Neotrop. Entomol..

[9] Zimmermann G (2007). Review on safety of the entomopathogenic fungus *Metarhizium anisopliae*. Biocontrol Sci. Technol..

[10] Alves, S. B. Fungos entomopatogênicos In *Controle microbiano de inseto* (ed. FEALQ) 289–382 (1998).

[11] Moura Mascarin, G. *et al*. Current status and perspectives of fungal entomopathogens used for microbial control of arthropod pests in Brazil. *J. Invertebr. Pathol*, 10.1016/j.jip.2018.01.001 (2018). 10.1016/j.jip.2018.01.001 29339191

[12] Parra J (2014). Biological control in Brazil. Sci. Agric..

[13] Torres de Carvalho LW (2010). Incidência de *Mahanarva fimbriolata* despues de aplicaciónes de *Metarhizium anisopliae* e imidacloprid en cana de azúcar. Revista Caatinga.

[14] Dinardo-Miranda L (2004). Eficiência de *Metarhizium anisopliae* (Metsch.) no Controle de *Mahanarva fimbriolata* (Stål) (Hemiptera: Cercopidae). Neotrop.Entomol..

[15] Kassab SO (2014). Combinations of *Metarhizium anisopliae* with chemical insecticides and their effectiveness in *Mahanarva fimbriolata* (Hemiptera: Cercopidae) control on sugarcane. Florida Entomol..

[16] Kassab, S. O. *et al*. Alteração no método de amostragem de *Mahanarva fimbriolata* (Stal,1854) (Hem.:Cercopidae) e avaliação da eficiência de *Metarhizium anisopliae* (Metschnikoff, 1879) Sorokin, 1883 (HYP.:Clavicipitaceae) *Arq. Inst. Biol*. **79**, 621–625 (2012).

[17] Tiago PV, Marcelo H, Souza DL, Moysés JB (2011). Differential Pathogenicity of *Metarhizium anisopliae* and the Control of the Sugarcane Root Spittlebug *Mahanarva fimbriolata*. Brazilian Arch. Biol. Technol..

[18] Velásquez VB, Cárcamo MP, Meriño CR, Iglesias AF, Durán JF (2007). Intraspecific differentiation of Chilean isolates of the entomopathogenic fungi *Metarhizium anisoplia*e var. *anisopliae* as revealed by RAPD, SSR and ITSmarkers. Reactions.

[19] Steinwender, B. M., Enkerli, J., Widmer, F. & Eilenberg, J. Archived at http://orgprints.org/19302 Molecular diversity of the *Metarhizium anisopliae* lineage in an agricultural field. *J. Appl. Entomol*. **66**, 113–115 (2011).

[20] Kepler RM, Ugine Ta, Maul JE, Cavigelli MA, Rehner SA (2015). Community composition and population genetics of insect pathogenic fungi in the genus *Metarhizium* from soils of a long-term agricultural research system. Environ. Microbiol..

[21] Rocha LFN, Inglis PW, Humber RA, Kipnis A, Luz C (2013). Occurrence of *Metarhizium* spp. in Central Brazilian soils. J. Basic Microbiol..

[22] Rocha LFN (2009). Occurrence of invertebrate-pathogenic fungi in a Cerrado ecosystem in Central Brazil. Biocontrol Sci. Technol..

[23] Kepler R, Humber R, Bischoff J, Rehner S (2014). Clarification of generic and species boundaries for *Metarhizium* and related fungi through multigene phylogenetics. Mycologia.

[24] Lopes RB, Souza DA, Oliveira CM, Faria M (2013). Genetic Diversity and Pathogenicity of *Metarhizium* spp. Associated with the White Grub *Phyllophaga capillata* (Blanchard) (Coleoptera: Melolonthidae) in a Soybean Field. Neotrop. Entomol..

[25] Lopes RB (2014). MALDI-TOF mass spectrometry applied to identifying species of insect-pathogenic fungi from the *Metarhizium anisopliae* complex. Mycologia.

[26] Lopes RB (2017). Metarhizium alvesii sp. nov.: A new member of the *Metarhizium anisopliae* species complex. J. Invertebr. Pathol..

[27] Rezende, J. M., Zanardo, A. B. R., da Silva Lopes, M., Delalibera, I. & Rehner, S. A. Phylogenetic diversity of Brazilian *Metarhizium* associated with sugarcane agriculture. *BioControl*, 10.1007/s10526-015-9656-5 (2015).

[28] Montalva C (2016). A natural fungal infection of a sylvatic cockroach with *Metarhizium blattodeae* sp. nov., a member of the *M. flavoviride* species complex. Fungal Biol..

[29] Hernández-Domínguez C, Guzmán-Franco AW (2017). Species Diversity and Population Dynamics of Entomopathogenic Fungal Species in the Genus *Metarhizium*—a Spatiotemporal Study. Microb. Ecol..

[30] Sasan RK, Bidochka MJ (2013). Antagonism of the endophytic insect pathogenic fungus *Metarhizium robertsii* against the bean plant pathogen Fusarium solani f. sp. phaseoli. Can. J. Plant Pathol..

[31] Sasan, R. K. & Bidochka, M. J. The insect-pathogenic fungus *Metarhizium robertsii* (Clavicipitaceae) is also an endophyte that stimulates plant root development. *Am J Bot* **99** (2012). 10.3732/ajb.1100136 22174335

[32] Elena GJ, Beatriz PJ, Alejandro P, E LR (2011). *Metarhizium anisopliae* (Metschnikoff) Sorokin Promotes Growth and Has Endophytic Activity in Tomato Plants. Adv. Biol. Res..

[33] Khan AL (2012). Pure culture of *Metarhizium anisopliae* LHL07 reprograms soybean to higher growth and mitigates salt stress. World J. Microbiol. Biotechnol..

[34] Behie SW, Zelisko PM, Bidochka MJ (2012). Endophytic Insect-Parasitic Fungi Translocate Nitrogen Directly from Insects to Plants. Science (80-.)..

[35] Behie, S. W. & Bidochka, M. J. Ubiquity of insect-derived nitrogen transfer to plants by endophytic insect-pathogenic fungi: an additional branch of the soil nitrogen cycle. *Appl Env. Microbiol* **80** (2014). 10.1128/AEM.03338-13 PMC3957595 24334669

[36] Castro T (2016). Persistence of Brazilian isolates of the entomopathogenic fungi *Metarhizium anisopliae* and *M. robertsii* in strawberry crop soil after soil drench application. Agric. Ecosyst. Environ..

[37] Pilz C, Enkerli J, Wegensteiner R, Keller S (2011). Establishment and persistence of the entomopathogenic fungus *Metarhizium anisopliae* in maize fields. J. Appl. Entomol..

[38] Roberts DW, St. Leger RJ (2004). Metarhizium spp., cosmopolitan insect-pathogenic fungi: Mycological aspects. Adv. Appl. Microbiol..

[39] Hernandez-Dominguez C (2016). Specific Diversity of *Metarhizium* Isolates Infecting Aeneolamia spp. (Hemiptera: Cercopidae) in Sugarcane Plantations. Neotrop. Entomol..

[40] Meyling NV, Thorup-Kristensen K, Eilenberg J (2011). Below- and aboveground abundance and distribution of fungal entomopathogens in experimental conventional and organic cropping systems. Biol. Control.

[41] Bruck DJ (2005). Ecology of *Metarhizium anisopliae* in soilless potting media and the rhizosphere: implications for pest management. Biol. Control.

[42] Pava-Ripoll M (2011). The rhizosphere-competent entomopathogen M*etarhizium anisopliae* expresses a specific subset of genes in plant root exudate. Microbiology.

[43] Wyrebek, M., Huber, C., Sasan, R. K. & Bidochka, M. J. Three sympatrically occurring species of *Metarhizium* show plant rhizosphere specificity. *Microbiology* **157** (2011). 10.1099/mic.0.051102-0 21778205

[44] Steinwender BM (2015). Root isolations of *Metarhizium* spp. from crops reflect diversity in the soil and indicate no plant specificity. J. Invertebr. Pathol..

[45] Spatafora JW, Sung GH, Sung HM, Hywel-Jones L, White JF (2007). Phylogenetic evidence for an animal pathogen origin of ergot and the grass endophytes. Mol. Ecol..

[46] Gao Q (2011). Genome sequencing and comparative transcriptomics of the model entomopathogenic fungi M*etarhizium anisopliae* and M*. acridum*. PLoS Genet..

[47] Barelli L, Moonjely S, Behie SW, Bidochka MJ (2016). Fungi with multifunctional lifestyles: endophytic insect pathogenic fungi. Plant. Mol. Biol..

[48] Kasambala Donga T, Vega FE, Klingen I (2018). Establishment of the fungal entomopathogen B*eauveria bassiana* as an endophyte in sugarcane, S*accharum officinarum*. Fungal Ecol..

[49] Steinwender BM (2014). Molecular diversity of the entomopathogenic fungal *Metarhizium* community within an agroecosystem. J. Invertebr. Pathol..

[50] Rehner SA, Kepler RM (2017). Species limits, phylogeography and reproductive mode in the *Metarhizium anisopliae* complex. J. Invertebr. Pathol..

[51] Zimmermann G (1986). The Galleria bait method for detection of entomopathogenic fungi in soil. J. of Appl. Entomol..

[52] Meyling NV, Eilenberg J (2006). Isolation and characterisation of B*eauveria bassiana* isolates from phylloplanes of hedgerow vegetation. Mycol. Res..

[53] Oulevey C, Widmer F, Kölliker R, Enkerli J (2009). An optimized microsatellite marker set for detection of *Metarhizium anisopliae* genotype diversity on field and regional scales. Mycol. Res..

[54] Enkerli J, Kölliker R, Keller S, Widmer F (2005). Isolation and characterization of microsatellite markers from the entomopathogenic fungus *Metarhizium anisopliae*. Mol. Ecol. Notes.

[55] Schuelke M (2000). An economic method for the fluorescent labeling of PCR fragments. Nat Biotechnol..

[56] Peakall R, Smouse PE (2012). GenALEx 6.5: Genetic analysis in Excel. Population genetic software for teaching and research-an update. Bioinformatics.

[57] Hurlbert, S. H. The Nonconcept of Species Diversity: A Critique and Alternative Parameters. Ecology 52 (4). *Eco Soc Americ*a: 577–86 (1971). 10.2307/1934145 28973811

[58] R Core Team R: A language and environment for statistical computing. R Foundation for Statistical Computing, Vienna, Austria, http://www.R-project.org/ (2017).

[59] Kamvar ZN, Tabima JF, Grünwald NJ (2014). *Poppr*: an R package for genetic analysis of populations with clonal, partially clonal, and/or sexual reproduction. PeerJ.

[60] Oksanen, J. *et al*. Stevens and Helene Wagner vegan: Community Ecology Package. R package version 2.3-0, https://CRAN.Rproject.org/package=vegan (2015).

[61] Brown AH, Feldman MW, Nevo E (1980). Multilocus Structure of Natural Populations of Hordeum Spontaneum. Genetics.

[62] Smith JM, Smith NH, O’Rourke M, Spratt BG (1993). How clonal are bacteria?. Proc. Natl. Acad. Sci..

[63] Agapow PM, Burt A (2001). Indices of multilocus linkage disequilibrium. Mol. Ecol. Notes.

[64] Bischoff JF, Rehner SA, Humber RA (2009). A multilocus phylogeny of the *Metarhizium anisopliae* lineage. Mycologia.

[65] Hall T (1999). BioEdit: a user-friendly biological sequence alignment editor and analysis program for Windows 95/98/NT. Nucleic Acids Symposium Series.

[66] Silvestro D, Michalak I (2012). RaxmlGUI: A graphical front-end for RAxML. Org. Divers. Evol..

[67] Stamatakis A, Hoover P, Rougemont J (2008). A rapid bootstrap algorithm for the RAxML Web servers. Syst. Biol..

[68] Tamura K, Stecher G, Peterson D, Filipski A, Kumar S (2013). MEGA6: Molecular evolutionary genetics analysis version 6.0. Mol. Biol. Evol..

[69] Bischoff JF, Rehner SA, Humber RA (2006). *Metarhizium frigidum* sp. nov.: a cryptic species of *M. anisopliae* and a member of the *M. flavoviride* complex. Mycologia.

[70] Rogers, J. S. Measures of genetic similarity and genetic distances. *Studies in Genetics*. 145–153 (1972).

