# Supplementary material for: Monitoring of the field application of Metarhizium anisopliae in Brazil revealed high molecular diversity of Metarhizium spp in insects, soil and sugarcane roots
Source: Sci Rep. 2019 Mar 14;9:4443. doi: 10.1038/s41598-019-38594-8 (PMC6418095; doi:10.1038/s41598-019-38594-8)
Supplement: Supplementary file 1 — Supplementary material [file 41598_2019_38594_MOESM1_ESM.docx]

**Monitoring of the field application of *M. anisopliae* in Brazil revealed high molecular diversity of *Metarhizium* spp in insects, soil and sugarcane roots.**

Natasha Sant´Anna Iwanicki, Alessandro Alves-Pereira, Ana Beatriz Riguetti Zanardo Botelho, Janayne Maria Rezende, Rafael de Andrade Moral, Maria Imaculada Zucchi, Italo Delalibera Júnior


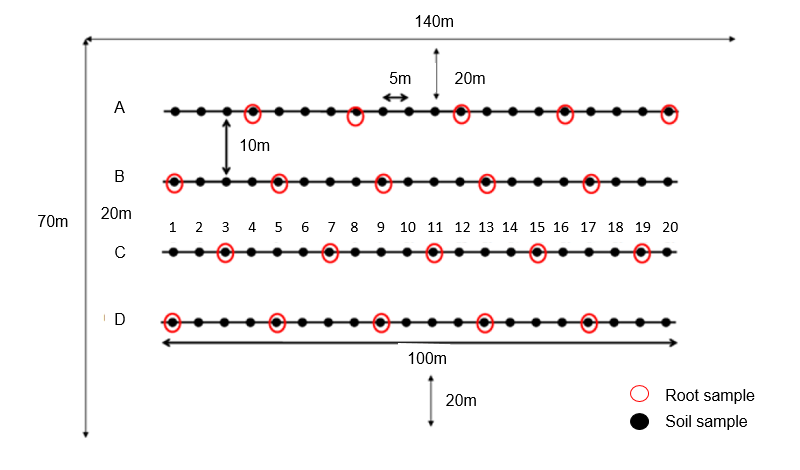


**Supplementary Fig. S1**. Scheme for soil and root sampling within sugarcane plot.


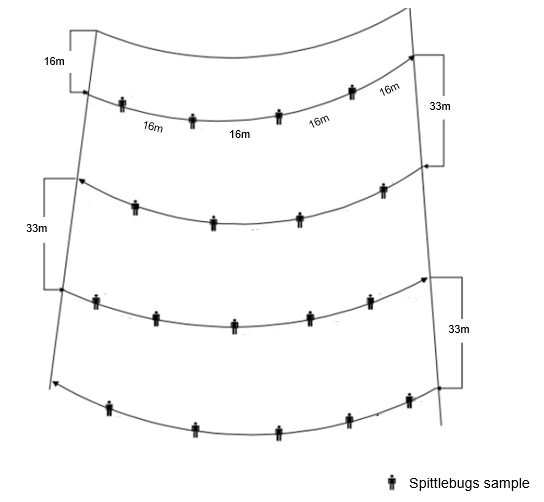


**Supplementary Fig. S2.** Scheme for spittlebug sampling in sugarcane plot.

**
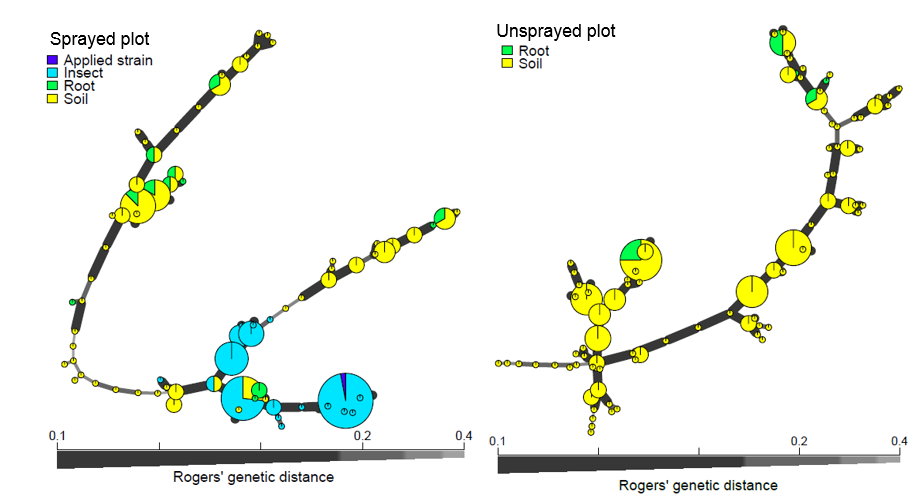
**

**Supplementary Fig. S3.** Minimum spanning network of multilocus microsatellite haplotypes (MMH) of *Metarhizium* spp. found in sprayed plot (n=166) and unsprayed plot (n=141). Circle sizes are proportional to the number of isolates recovered for each MMH. The applied strain is ESALQ1604. The width of lines between circles indicates Rogers’ genetic distance**^70^**. Different colors indicate the origin of isolates (insect, soil, root and applied strain).

**Supplementary Table S1: Number of adults and nymphs of spittlebugs collected by sampling date and recovered isolates.**

| Before application | Days before and after fungus´s application | Sampling date | N° of Adults | N° of Isolates | N° of Nymphs | N° of Isolates | Total |
| --- | --- | --- | --- | --- | --- | --- | --- |
|  | 51 | November 19^th^ , 2014 | 22 | 4 | 54 | 8 | 76 |
|  | 42 | November 28^th^ , 2014 | 41 | 3 | 17 | 0 | 58 |
|  | 1 | January 08^th^ , 2015 | 16 | 0 | 21 | 0 | 37 |
| After  application | 7 | January 16^th^ , 2015 | 28 | 3 | 97 | 49 | 125 |
|  | 30 | February 12^th ,^2015 | 31 | 2 | 31 | 0 | 62 |
|  | 60 | March 12^th ,^2015 | 12 | 2 | 90 | 20 | 102 |
|  | 90 | April 15^th ,^2015 | 15 | 0 | 19 | 0 | 34 |

**Supplementary Table S2. Multilocus microsatellite haplotype, microsatellites (fluorescence), origin of sample, collected date, plot (1=sprayed plot; 2=unsprayed plot).** The MMH 130 and 152 represent ESALQ 5310 (sugarcane mill strain (SM)) and ESALQ 1604 (the applied strain (AS) in this study) respectively.

| MMH | 2097  (FAM) | 142  (HEX) | 2054  (HEX) | 2099  (NED) | 2065  (FAM) | 2064  (HEX) | 165  (NED) | 2292  (FAM) | 2296  (HEX) | 2063  (NED) | ORIGIN | DATE | PLOT | N° of Isolates | Representative isolate |
| --- | --- | --- | --- | --- | --- | --- | --- | --- | --- | --- | --- | --- | --- | --- | --- |
| 152 | 214 | 127 | 242 | 266 | 147 | 172 | 160 | 216 | 160 | 163 | AS, Insect | 7D, 30D, 60D | 1 | 33 | ESALQ1604 |
| 11 | 204 | 136 | 236 | 264 | 147 | 172 | 156 | 216 | 160 | 165 | soil | 2012 | 2 | 1 | ESALQ1652 |
| 14 | 204 | 142 | 240 | 264 | 147 | 172 | 160 | 216 | 160 | 155 | soil | 2012 | 2 | 1 | ESALQ1658 |
| 131 | 204 | 124 | 252 | 262 | 147 | 172 | 156 | 216 | 160 | 165 | soil | 2012 | 2 | 2 | ESALQ1661 |
| 139 | 206 | 142 | 240 | 264 | 147 | 172 | 160 | 216 | 160 | 155 | soil | 2012 | 1 , 2 | 2 | ESALQ1664 |
| 15 | 204 | 142 | 240 | 264 | 147 | 172 | 160 | 216 | 160 | 157 | soil | 2012 | 2 | 1 | ESALQ1665 |
| 20 | 205 | 124 | 252 | 262 | 147 | 172 | 156 | 216 | 160 | 165 | soil | 2012 | 2 | 1 | ESALQ1668 |
| 50 | 207 | 124 | 240 | 264 | 147 | 172 | 160 | 216 | 160 | 155 | soil | 2012 | 2 | 1 | ESALQ1670 |
| 153 | 206 | 144 | 240 | 264 | 147 | 172 | 160 | 216 | 160 | 155 | soil | 2012 | 2 | 2 | ESALQ1673 |
| 8 | 204 | 127 | 242 | 266 | 147 | 172 | 160 | 216 | 162 | 157 | soil | 2012 | 2 | 1 | ESALQ1683 |
| 109 | 214 | 142 | 242 | 266 | 147 | 172 | 156 | 216 | 160 | 163 | soil | PA | 2 | 1 | ESALQ4978 |
| 29 | 205 | 136 | 236 | 268 | 145 | 182 | 160 | 216 | 160 | 161 | soil | PA | 2 | 1 | ESALQ4992 |
| 74 | 207 | 143 | 240 | 266 | 145 | 182 | 160 | 216 | 160 | 161 | soil | PA | 2 | 1 | ESALQ4993 |
| 132 | 214 | 124 | 262 | 266 | 145 | 178 | 156 | 216 | 170 | 169 | soil | PA | 1 | 2 | ESALQ4999 |
| 146 | 214 | 127 | 242 | 266 | 147 | 172 | 156 | 216 | 162 | 163 | insect, soil | PA | 1 , 2 | 3 | ESALQ5001 |
| 27 | 205 | 125 | 252 | 264 | 147 | 182 | 160 | 201 | 160 | 161 | soil | PA | 1 | 1 | ESALQ5032 |
| 76 | 207 | 144 | 240 | 266 | 145 | 182 | 160 | 216 | 160 | 155 | soil | PA | 2 | 1 | ESALQ5060 |
| 140 | 214 | 127 | 242 | 266 | 147 | 172 | 156 | 216 | 160 | 163 | insect, root | PA, 30D, 90D | 1 , 2 | 19 | ESALQ5077 |
| 102 | 214 | 127 | 242 | 266 | 147 | 172 | 160 | 216 | 170 | 163 | insect | 7D | 1 | 1 | ESALQ5120 |
| 64 | 207 | 142 | 240 | 264 | 183 | 182 | 160 | 216 | 160 | 155 | soil | 30D | 2 | 1 | ESALQ5172 |
| 143 | 206 | 143 | 240 | 264 | 145 | 182 | 160 | 216 | 160 | 155 | soil | PA, 90D | 2 | 4 | ESALQ5178 |
| 4 | 204 | 124 | 252 | 262 | 147 | 182 | 156 | 216 | 160 | 165 | soil | PA | 2 | 1 | ESALQ5179 |
| 171 | 214 | 124 | 262 | 264 | 145 | 178 | 156 | 216 | 170 | 169 | root and soil | PA, 30D | 1 | 2 | ESALQ5181 |
| 18 | 204 | 143 | 242 | 264 | 145 | 182 | 160 | 216 | 164 | 155 | soil | PA | 2 | 1 | ESALQ5240 |
| 154 | 214 | 127 | 242 | 266 | 147 | 172 | 160 | 216 | 162 | 163 | insect | 7D, 60D | 1 | 7 | ESALQ5244 |
| 108 | 214 | 128 | 242 | 266 | 147 | 172 | 160 | 216 | 160 | 163 | insect | 60D | 1 | 1 | ESALQ5245 |
| 133 | 205 | 142 | 240 | 264 | 147 | 182 | 160 | 216 | 160 | 155 | soil | 30D | 1 , 2 | 5 | ESALQ5247 |
| 168 | 214 | 124 | 262 | 262 | 145 | 178 | 156 | 216 | 170 | 147 | root and soil | 30D, PA | 1 | 8 | ESALQ5249 |
| 155 | 207 | 142 | 240 | 264 | 145 | 172 | 160 | 216 | 160 | 155 | soil | 2012, PA, 30D | 1 , 2 | 4 | ESALQ5250 |
| 104 | 214 | 127 | 242 | 266 | 147 | 182 | 160 | 216 | 164 | 163 | insect | 7D | 1 | 1 | ESALQ5251 |
| 22 | 205 | 124 | 252 | 262 | 183 | 182 | 156 | 216 | 160 | 165 | soil | 30D | 1 | 1 | ESALQ5252 |
| 17 | 204 | 142 | 248 | 264 | 183 | 182 | 160 | 216 | 160 | 161 | soil | 30D | 2 | 1 | ESALQ5253 |
| 149 | 206 | 143 | 240 | 264 | 183 | 182 | 160 | 216 | 160 | 161 | root, soil | 30D | 1 , 2 | 15 | ESALQ5257 |
| 13 | 204 | 142 | 240 | 264 | 147 | 172 | 156 | 216 | 160 | 165 | soil | 30D | 2 | 1 | ESALQ5258 |
| 137 | 206 | 142 | 240 | 264 | 145 | 172 | 160 | 216 | 160 | 155 | soil | 2012,30D, 90D | 1 , 2 | 10 | ESALQ5263 |
| 19 | 205 | 124 | 252 | 262 | 147 | 172 | 156 | 201 | 160 | 165 | soil | 30D | 2 | 1 | ESALQ5264 |
| 63 | 207 | 142 | 240 | 264 | 155 | 182 | 160 | 216 | 160 | 155 | soil | 30D | 2 | 1 | ESALQ5265 |
| 60 | 207 | 142 | 240 | 264 | 145 | 182 | 160 | 216 | 160 | 161 | soil | 30D | 2 | 1 | ESALQ5266 |
| 58 | 207 | 142 | 240 | 264 | 145 | 172 | 160 | 216 | 160 | 163 | soil | 30D | 2 | 1 | ESALQ5268 |
| 66 | 207 | 143 | 240 | 264 | 145 | 172 | 160 | 216 | 160 | 161 | soil | 90D | 2 | 1 | ESALQ5276 |
| 145 | 206 | 143 | 240 | 264 | 145 | 182 | 160 | 216 | 160 | 161 | soil | 90D | 2 | 3 | ESALQ5277 |
| 45 | 206 | 143 | 240 | 264 | 183 | 178 | 160 | 216 | 160 | 161 | soil | 90D | 2 | 1 | ESALQ5278 |
| 141 | 206 | 143 | 240 | 264 | 145 | 172 | 160 | 216 | 160 | 161 | soil | 90D | 2 | 3 | ESALQ5279 |
| 38 | 206 | 124 | 252 | 262 | 145 | 172 | 160 | 216 | 160 | 155 | soil | 90D | 2 | 1 | ESALQ5280 |
| 10 | 204 | 136 | 236 | 262 | 145 | 172 | 160 | 216 | 160 | 155 | soil | 90D | 2 | 1 | ESALQ5281 |
| 40 | 206 | 142 | 240 | 264 | 145 | 172 | 160 | 216 | 160 | 161 | soil | 90D | 1 | 1 | ESALQ5283 |
| 9 | 204 | 127 | 252 | 264 | 147 | 172 | 156 | 216 | 160 | 165 | soil | 90D | 1 | 1 | ESALQ5284 |
| 36 | 206 | 124 | 240 | 264 | 145 | 172 | 160 | 216 | 160 | 163 | soil | 90D | 1 | 1 | ESALQ5285 |
| 82 | 214 | 124 | 262 | 264 | 145 | 178 | 156 | 216 | 160 | 147 | soil | 90D | 1 | 1 | ESALQ5286 |
| 138 | 214 | 127 | 242 | 266 | 147 | 172 | 156 | 216 | 160 | 161 | root and soil | PA | 2 | 3 | ESALQ5289 |
| 165 | 207 | 143 | 240 | 264 | 145 | 182 | 160 | 216 | 160 | 161 | soil | PA, 90D | 1 , 2 | 8 | ESALQ5290 |
| 130 | 214 | 128 | 244 | 266 | 145 | 172 | 158 | 216 | 162 | 155 | SM |  | 1 | 1 | ESALQ5310 |
| 159 | 207 | 142 | 240 | 264 | 147 | 172 | 160 | 216 | 162 | 155 | soil | PA | 2 | 2 | ESALQ5320 |
| 31 | 205 | 136 | 236 | 270 | 145 | 172 | 160 | 216 | 162 | 155 | soil | PA | 2 | 1 | ESALQ5321 |
| 59 | 207 | 142 | 240 | 264 | 145 | 172 | 160 | 216 | 162 | 147 | soil | PA | 2 | 1 | ESALQ5322 |
| 167 | 207 | 143 | 240 | 264 | 145 | 182 | 160 | 216 | 162 | 161 | soil | PA | 2 | 2 | ESALQ5323 |
| 67 | 207 | 143 | 240 | 264 | 145 | 172 | 160 | 216 | 162 | 155 | soil | PA | 2 | 1 | ESALQ5324 |
| 98 | 214 | 127 | 242 | 266 | 147 | 172 | 156 | 216 | 164 | 155 | soil | PA | 2 | 1 | ESALQ5325 |
| 142 | 214 | 127 | 242 | 266 | 147 | 172 | 156 | 216 | 162 | 147 | soil | PA | 2 | 2 | ESALQ5326 |
| 65 | 207 | 142 | 240 | 266 | 147 | 172 | 160 | 216 | 162 | 155 | soil | PA | 2 | 1 | ESALQ5327 |
| 166 | 207 | 143 | 240 | 264 | 145 | 182 | 160 | 216 | 162 | 155 | soil | PA | 2 | 2 | ESALQ5328 |
| 49 | 207 | 124 | 240 | 264 | 145 | 172 | 160 | 216 | 160 | 161 | soil | PA | 2 | 1 | ESALQ5329 |
| 34 | 205 | 142 | 240 | 264 | 147 | 182 | 160 | 216 | 162 | 155 | soil | PA | 2 | 1 | ESALQ5331 |
| 144 | 214 | 127 | 242 | 266 | 147 | 172 | 156 | 216 | 162 | 161 | soil | PA, 30D | 1 | 3 | ESALQ5332 |
| 92 | 214 | 124 | 262 | 266 | 145 | 178 | 158 | 216 | 170 | 147 | root | PA | 1 | 1 | ESALQ5333 |
| 23 | 205 | 124 | 252 | 264 | 145 | 178 | 156 | 201 | 160 | 169 | soil | PA | 1 | 1 | ESALQ5334 |
| 7 | 204 | 124 | 252 | 264 | 147 | 182 | 156 | 201 | 160 | 165 | soil | PA | 1 | 1 | ESALQ5335 |
| 81 | 214 | 124 | 262 | 262 | 147 | 182 | 156 | 216 | 170 | 169 | soil | PA | 1 | 1 | ESALQ5336 |
| 25 | 205 | 124 | 252 | 264 | 147 | 182 | 156 | 216 | 162 | 165 | soil | PA | 1 | 1 | ESALQ5337 |
| 173 | 214 | 124 | 262 | 266 | 145 | 178 | 156 | 216 | 170 | 147 | root and soil | PA | 1 | 6 | ESALQ5338 |
| 26 | 205 | 125 | 252 | 264 | 147 | 182 | 156 | 201 | 160 | 165 | soil | PA | 1 | 1 | ESALQ5339 |
| 78 | 214 | 124 | 262 | 262 | 145 | 178 | 156 | 216 | 170 | 163 | soil | PA | 1 | 1 | ESALQ5340 |
| 53 | 207 | 127 | 240 | 264 | 147 | 172 | 160 | 216 | 162 | 155 | soil | PA | 1 | 1 | ESALQ5341 |
| 97 | 214 | 127 | 242 | 266 | 147 | 172 | 156 | 216 | 160 | 147 | soil | PA | 1 | 1 | ESALQ5342 |
| 77 | 214 | 124 | 262 | 262 | 145 | 178 | 156 | 216 | 170 | 161 | soil | PA | 1 | 1 | ESALQ5343 |
| 163 | 207 | 143 | 240 | 264 | 145 | 182 | 160 | 216 | 160 | 155 | soil | PA | 1 , 2 | 4 | ESALQ5344 |
| 169 | 214 | 124 | 262 | 262 | 145 | 178 | 156 | 216 | 170 | 169 | soil | PA | 1 | 2 | ESALQ5345 |
| 51 | 207 | 124 | 262 | 264 | 145 | 178 | 156 | 216 | 170 | 147 | soil | PA | 1 | 1 | ESALQ5346 |
| 170 | 214 | 124 | 262 | 264 | 145 | 178 | 156 | 216 | 170 | 147 | soil | PA | 1 | 2 | ESALQ5347 |
| 87 | 214 | 124 | 262 | 264 | 147 | 178 | 156 | 216 | 170 | 169 | soil | PA | 1 | 1 | ESALQ5348 |
| 55 | 207 | 128 | 240 | 264 | 145 | 172 | 160 | 216 | 160 | 161 | soil | PA | 2 | 1 | ESALQ5349 |
| 30 | 205 | 136 | 236 | 270 | 145 | 172 | 160 | 216 | 160 | 161 | soil | PA | 2 | 1 | ESALQ5350 |
| 100 | 214 | 127 | 242 | 266 | 147 | 172 | 158 | 216 | 162 | 161 | soil | PA | 2 | 1 | ESALQ5351 |
| 56 | 207 | 128 | 240 | 264 | 145 | 182 | 160 | 216 | 160 | 155 | soil | PA | 2 | 1 | ESALQ5352 |
| 99 | 214 | 127 | 242 | 266 | 147 | 172 | 156 | 216 | 164 | 165 | root | PA | 2 | 1 | ESALQ5353 |
| 107 | 214 | 128 | 242 | 266 | 147 | 172 | 158 | 216 | 160 | 163 | insect | PA | 1 | 1 | ESALQ5354 |
| 71 | 207 | 143 | 240 | 264 | 147 | 182 | 160 | 216 | 162 | 155 | soil | 30D | 2 | 1 | ESALQ5355 |
| 24 | 205 | 124 | 252 | 264 | 147 | 172 | 160 | 216 | 162 | 165 | soil | 30D | 2 | 1 | ESALQ5356 |
| 101 | 214 | 127 | 242 | 266 | 147 | 172 | 160 | 216 | 164 | 163 | soil | 30D | 2 | 1 | ESALQ5357 |
| 75 | 207 | 143 | 242 | 264 | 147 | 172 | 160 | 216 | 160 | 147 | insect | 7D | 1 | 1 | ESALQ5358 |
| 57 | 207 | 128 | 242 | 266 | 147 | 172 | 160 | 216 | 160 | 163 | insect | 7D | 1 | 1 | ESALQ5359 |
| 54 | 207 | 127 | 242 | 264 | 147 | 182 | 160 | 216 | 162 | 163 | insect | 7D | 1 | 1 | ESALQ5360 |
| 156 | 214 | 127 | 242 | 266 | 147 | 178 | 160 | 216 | 162 | 163 | insect | 7D | 1 | 3 | ESALQ5361 |
| 103 | 214 | 127 | 242 | 266 | 147 | 182 | 160 | 216 | 162 | 161 | insect | 7D | 1 | 1 | ESALQ5362 |
| 158 | 214 | 127 | 242 | 266 | 147 | 182 | 160 | 216 | 162 | 163 | insect | 7D | 1 | 4 | ESALQ5363 |
| 96 | 214 | 127 | 242 | 264 | 147 | 182 | 158 | 216 | 160 | 163 | insect | 30D | 1 | 1 | ESALQ5364 |
| 47 | 206 | 144 | 240 | 264 | 145 | 172 | 160 | 216 | 160 | 155 | soil | PA | 2 | 1 | ESALQ5365 |
| 79 | 214 | 124 | 262 | 262 | 147 | 178 | 156 | 216 | 160 | 147 | soil | 30D | 1 | 1 | ESALQ5366 |
| 90 | 214 | 124 | 262 | 264 | 177 | 178 | 156 | 216 | 170 | 163 | soil | 30D | 1 | 1 | ESALQ5367 |
| 89 | 214 | 124 | 262 | 264 | 177 | 178 | 156 | 216 | 162 | 147 | soil | 30D | 1 | 1 | ESALQ5368 |
| 70 | 207 | 143 | 240 | 264 | 147 | 182 | 160 | 216 | 160 | 161 | root | 30D | 1 | 1 | ESALQ5369 |
| 80 | 214 | 124 | 262 | 262 | 147 | 178 | 156 | 216 | 160 | 161 | soil | 30D | 1 | 1 | ESALQ5370 |
| 46 | 206 | 143 | 240 | 264 | 183 | 182 | 160 | 216 | 160 | 155 | soil | 30D | 1 | 1 | ESALQ5371 |
| 91 | 214 | 124 | 262 | 264 | 177 | 178 | 156 | 216 | 170 | 169 | soil | 30D | 1 | 1 | ESALQ5372 |
| 160 | 214 | 127 | 242 | 266 | 171 | 172 | 156 | 216 | 160 | 161 | root | 30D | 1 | 2 | ESALQ5373 |
| 52 | 207 | 127 | 240 | 264 | 145 | 182 | 160 | 216 | 160 | 155 | soil | 30D | 2 | 1 | ESALQ5374 |
| 32 | 205 | 140 | 252 | 264 | 183 | 182 | 160 | 216 | 162 | 155 | soil | 30D | 2 | 1 | ESALQ5375 |
| 69 | 207 | 143 | 240 | 264 | 147 | 172 | 156 | 216 | 162 | 155 | soil | 30D | 2 | 1 | ESALQ5376 |
| 151 | 206 | 143 | 240 | 264 | 183 | 182 | 160 | 216 | 162 | 155 | soil | 30D | 2 | 2 | ESALQ5377 |
| 41 | 206 | 142 | 240 | 264 | 145 | 172 | 160 | 216 | 164 | 155 | soil | 30D | 2 | 1 | ESALQ5378 |
| 16 | 204 | 142 | 240 | 264 | 147 | 182 | 160 | 216 | 160 | 161 | soil | 30D | 2 | 1 | ESALQ5379 |
| 73 | 207 | 143 | 240 | 264 | 183 | 182 | 160 | 216 | 160 | 155 | soil | 30D | 2 | 1 | ESALQ5380 |
| 33 | 205 | 142 | 240 | 264 | 147 | 182 | 160 | 216 | 160 | 159 | soil | 30D | 2 | 1 | ESALQ5381 |
| 1 | 202 | 136 | 234 | 264 | 145 | 182 | 164 | 216 | 160 | 161 | soil | 30D | 2 | 1 | ESALQ5382 |
| 12 | 204 | 136 | 236 | 264 | 183 | 172 | 160 | 216 | 160 | 161 | soil | 30D | 2 | 1 | ESALQ5383 |
| 28 | 205 | 136 | 236 | 264 | 145 | 182 | 160 | 216 | 160 | 155 | soil | 30D | 2 | 1 | ESALQ5384 |
| 68 | 207 | 143 | 240 | 264 | 145 | 182 | 160 | 216 | 164 | 163 | soil | 30D | 2 | 1 | ESALQ5385 |
| 95 | 214 | 127 | 242 | 264 | 147 | 182 | 156 | 216 | 160 | 161 | insect | 60D | 1 | 1 | ESALQ5386 |
| 172 | 214 | 124 | 262 | 264 | 177 | 178 | 156 | 216 | 170 | 147 | root and soil | 30D | 1 | 3 | ESALQ5387 |
| 147 | 206 | 143 | 240 | 264 | 171 | 182 | 160 | 216 | 160 | 161 | soil | 30D, 90D | 2 | 2 | ESALQ5388 |
| 42 | 206 | 143 | 240 | 264 | 147 | 172 | 160 | 216 | 160 | 161 | soil | 90D | 2 | 1 | ESALQ5389 |
| 43 | 206 | 143 | 240 | 264 | 171 | 172 | 160 | 216 | 160 | 155 | soil | 90D | 2 | 1 | ESALQ5390 |
| 44 | 206 | 143 | 240 | 264 | 183 | 172 | 160 | 216 | 160 | 161 | soil | 90D | 2 | 1 | ESALQ5391 |
| 35 | 206 | 119 | 250 | 264 | 143 | 172 | 158 | 216 | 164 | 163 | soil | 90D | 2 | 1 | ESALQ5392 |
| 157 | 207 | 142 | 240 | 264 | 145 | 182 | 160 | 216 | 160 | 155 | soil | 30D, 90D | 2 | 6 | ESALQ5393 |
| 21 | 205 | 124 | 252 | 262 | 147 | 182 | 156 | 201 | 160 | 161 | soil | 90D | 1 | 1 | ESALQ5394 |
| 83 | 214 | 124 | 262 | 264 | 145 | 178 | 156 | 216 | 160 | 155 | soil | 90D | 1 | 1 | ESALQ5395 |
| 164 | 214 | 128 | 242 | 266 | 147 | 172 | 156 | 216 | 162 | 165 | root and soil | PA | 1 , 2 | 2 |  |
| 162 | 214 | 127 | 242 | 266 | 171 | 172 | 156 | 216 | 160 | 163 | root and soil | 30D | 1 , 2 | 2 |  |
| 161 | 207 | 142 | 240 | 264 | 147 | 182 | 160 | 216 | 160 | 155 | soil | 30D, 90D | 1 , 2 | 2 |  |
| 150 | 214 | 127 | 242 | 266 | 147 | 172 | 158 | 216 | 160 | 163 | insect | PA, 60D | 1 | 2 |  |
| 148 | 214 | 127 | 242 | 266 | 147 | 172 | 156 | 216 | 162 | 165 | soil | PA | 1 | 2 |  |
| 136 | 214 | 124 | 262 | 266 | 177 | 178 | 156 | 216 | 170 | 147 | root and soil | PA | 1 | 2 |  |
| 135 | 206 | 124 | 240 | 264 | 147 | 172 | 160 | 216 | 160 | 155 | soil | 2012, 90D | 2 | 2 |  |
| 134 | 214 | 124 | 262 | 266 | 147 | 178 | 156 | 216 | 170 | 147 | root and soil | PA | 1 | 2 |  |
| 106 | 214 | 128 | 242 | 266 | 147 | 172 | 156 | 216 | 162 | 163 | insect | PA | 1 | 1 |  |
| 105 | 214 | 127 | 242 | 266 | 171 | 172 | 156 | 216 | 164 | 163 | soil | 30D | 1 | 1 |  |
| 94 | 214 | 125 | 262 | 262 | 145 | 178 | 156 | 216 | 170 | 147 | soil | PA | 1 | 1 |  |
| 93 | 214 | 124 | 262 | 266 | 177 | 178 | 156 | 216 | 160 | 147 | root | 30D | 1 | 1 |  |
| 88 | 214 | 124 | 262 | 264 | 177 | 178 | 156 | 216 | 160 | 161 | root | 30D | 1 | 1 |  |
| 86 | 214 | 124 | 262 | 264 | 147 | 178 | 156 | 214 | 170 | 169 | soil | PA | 1 | 1 |  |
| 85 | 214 | 124 | 262 | 264 | 145 | 178 | 158 | 216 | 160 | 147 | soil | 90D | 1 | 1 |  |
| 84 | 214 | 124 | 262 | 264 | 145 | 178 | 156 | 216 | 164 | 147 | soil | 90D | 1 | 1 |  |
| 72 | 207 | 143 | 240 | 264 | 183 | 182 | 156 | 216 | 160 | 161 | soil | 30D | 2 | 1 |  |
| 62 | 207 | 142 | 240 | 264 | 147 | 182 | 156 | 216 | 160 | 155 | soil | 30D | 1 | 1 |  |
| 61 | 207 | 142 | 240 | 264 | 147 | 172 | 156 | 216 | 160 | 161 | soil | PA | 2 | 1 |  |
| 48 | 207 | 119 | 248 | 264 | 147 | 182 | 160 | 216 | 172 | 163 | soil | 30D | 2 | 1 |  |
| 39 | 206 | 124 | 262 | 264 | 147 | 172 | 160 | 216 | 160 | 163 | soil | 2012 | 2 | 1 |  |
| 37 | 206 | 124 | 240 | 264 | 147 | 182 | 160 | 216 | 160 | 155 | soil | 2012 | 2 | 1 |  |
| 6 | 204 | 124 | 252 | 264 | 147 | 172 | 156 | 216 | 160 | 165 | soil | 2012 | 2 | 1 |  |
| 5 | 204 | 124 | 252 | 262 | 147 | 182 | 162 | 216 | 160 | 147 | soil | 90D | 1 | 1 |  |
| 3 | 204 | 124 | 252 | 262 | 147 | 172 | 156 | 216 | 160 | 163 | soil | 2012 | 2 | 1 |  |
| 2 | 204 | 119 | 240 | 264 | 147 | 182 | 158 | 216 | 164 | 163 | soil | PA | 2 | 1 |  |

**Supplementary Table S3. *Metarhizium* strains used for reconstruction of Maximum likelihood phylogeny of 5´-TEF. Voucher, species, isolation source, GenBank accession numbers and Multilocus microsatellite haplotype (MMH)**

| Voucher | Species | Isolation_source | GenBank accession numbers | MMH |
| --- | --- | --- | --- | --- |
| ESALQ_5338 | *M. brunneum* | soil | MH596831 | **173** |
| ESALQ_5387 | *M. brunneum* | soil | MH596844 | **172** |
| ESALQ_5181 | *M. brunneum* | root | MH596826 | **171** |
| ESALQ_5347 | *M. brunneum* | soil | MH596837 | **170** |
| ESALQ_5345 | *M. brunneum* | soil | MH596835 | **169** |
| ESALQ_5249 | *M. brunneum* | root | MH596827 | **168** |
| ESALQ_5323 | *M. robertsii* | soil | MH596792 | **167** |
| ESALQ_5328 | *M. robertsii* | soil | MH596847 | **166** |
| ESALQ_5290 | *M. robertsii* | soil | MH596788 | **165** |
| ESALQ_5344 | *M. robertsii* | soil | MH596818 | **163** |
| ESALQ_5373 | *M. anisopliae* | root | MH596759 | **160** |
| ESALQ_5320 | *M. robertsii* | soil | MH596789 | **159** |
| ESALQ_5363 | *M. anisopliae* | insect | MH596752 | **158** |
| ESALQ_5393 | *M. robertsii* | soil | MH596794 | **157** |
| ESALQ_5361 | *M. anisopliae* | insect | MH596750 | **156** |
| ESALQ_5250 | *M. robertsii* | soil | MH596773 | **155** |
| ESALQ_5244 | *M. anisopliae* | insect | MH596757 | **154** |
| ESALQ_1673 | *M. robertsii* | soil | MH596721 | **153** |
| ESALQ_1604 | *M. anisopliae* |  | MH596728 | **152** |
| ESALQ_5163 | *M. anisopliae* | insect | MH596754 | **152** |
| ESALQ_5377 | *M. robertsii* | soil | MH596805 | **151** |
| ESALQ_5257 | *M. robertsii* | soil | MH596775 | **149** |
| ESALQ_5388 | *M. robertsii* | soil | MH596797 | **147** |
| ESALQ_5001 | *M. anisopliae* | soil | MH596739 | **146** |
| ESALQ_5277 | *M. robertsii* | soil | MH596781 | **145** |
| ESALQ_5332 | *M. anisopliae* | soil | MH596733 | **144** |
| ESALQ_5178 | *M. robertsii* | soil | MH596770 | **143** |
| ESALQ_5326 | *M. anisopliae* | soil | MH596730 | **142** |
| ESALQ_5279 | *M. robertsii* | soil | MH596783 | **141** |
| ESALQ_5077 | *M. anisopliae* | soil | MH596741 | **140** |
| ESALQ_1664 | *M. robertsii* | soil | MH596720 | **139** |
| ESALQ_5289 | *M. anisopliae* | root | MH596732 | **138** |
| ESALQ_5263 | *M. robertsii* | soil | MH596776 | **137** |
| ESALQ_5247 | *M. robertsii* | soil | MH596772 | **133** |
| ESALQ_4999 | *M. brunneum* | soil | MH596825 | **132** |
| ESALQ_1661 | *M. robertsii* | soil | MH596719 | **131** |
| ESALQ_5353 | *M. anisopliae* | root | MH596742 | **99** |
| ESALQ_5325 | *M. anisopliae* | soil | MH596729 | **98** |
| ESALQ_5342 | *M. anisopliae* | soil | MH596738 | **97** |
| ESALQ_5364 | *M. anisopliae* | insect | MH596755 | **96** |
| ESALQ_5386 | *M. anisopliae* | insect | MH596763 | **95** |
| ESALQ_5333 | *M. brunneum* | root | MH596830 | **92** |
| ESALQ_5372 | *M. brunneum* | soil | MH596843 | **91** |
| ESALQ_5367 | *M. brunneum* | soil | MH596840 | **90** |
| ESALQ_5284 | *M. anisopliae* | soil | MH596764 | **9** |
| ESALQ_5368 | *M. brunneum* | soil | MH596841 | **89** |
| ESALQ_5348 | *M. brunneum* | soil | MH596838 | **87** |
| ESALQ_5395 | *M. brunneum* | soil | MH596845 | **83** |
| ESALQ_5286 | *M. brunneum* | soil | MH596828 | **82** |
| ESALQ_5336 | *M. brunneum* | soil | MH596829 | **81** |
| ESALQ_5370 | *M. brunneum* | soil | MH596842 | **80** |
| ESALQ_1683 | *M. robertsii* | soil | MH596724 | **8** |
| ESALQ_5366 | *M. brunneum* | soil | MH596839 | **79** |
| ESALQ_5340 | *M. brunneum* | soil | MH596833 | **78** |
| ESALQ_5343 | *M. brunneum* | soil | MH596834 | **77** |
| ESALQ_5060 | *M. robertsii* | soil | MH596768 | **76** |
| ESALQ_5358 | *M. anisopliae* | insect | MH596746 | **75** |
| ESALQ_4993 | *M. robertsii* | soil | MH596767 | **74** |
| ESALQ_5380 | *M. robertsii* | soil | MH596801 | **73** |
| ESALQ_5355 | *M. robertsii* | soil | MH596814 | **71** |
| ESALQ_5369 | *M. robertsii* | soil | MH596812 | **70** |
| ESALQ_5335 | *M. anisopliae* | soil | MH596735 | **7** |
| ESALQ_5376 | *M. robertsii* | soil | MH596808 | **69** |
| ESALQ_5385 | *M. robertsii* | soil | MH596799 | **68** |
| ESALQ_5324 | *M. robertsii* | soil | MH596793 | **67** |
| ESALQ_5276 | *M. robertsii* | soil | MH596780 | **66** |
| ESALQ_5327 | *M. robertsii* | soil | MH596823 | **65** |
| ESALQ_5172 | *M. robertsii* | soil | MH596769 | **64** |
| ESALQ_5265 | *M. robertsii* | soil | MH596777 | **63** |
| ESALQ_5266 | *M. robertsii* | soil | MH596778 | **60** |
| ESALQ_5322 | *M. robertsii* | soil | MH596791 | **59** |
| ESALQ_5268 | *M. robertsii* | soil | MH596779 | **58** |
| ESALQ_5359 | *M. anisopliae* | insect | MH596747 | **57** |
| ESALQ_5352 | *M. robertsii* | soil | MH596815 | **56** |
| ESALQ_5349 | *M. robertsii* | soil | MH596817 | **55** |
| ESALQ_5360 | *M. anisopliae* | insect | MH596748 | **54** |
| ESALQ_5341 | *M. robertsii* | soil | MH596819 | **53** |
| ESALQ_5374 | *M. robertsii* | soil | MH596810 | **52** |
| ESALQ_5346 | *M. brunneum* | soil | MH596836 | **51** |
| ESALQ_1670 | *M. anisopliae* | soil | MH596713 | **50** |
| ESALQ_5329 | *M. robertsii* | soil | MH596822 | **49** |
| ESALQ_5365 | *M. robertsii* | soil | MH596813 | **47** |
| ESALQ_5371 | *M. robertsii* | soil | MH596811 | **46** |
| ESALQ_5278 | *M. robertsii* | soil | MH596782 | **45** |
| ESALQ_5391 | *M. robertsii* | soil | MH596796 | **44** |
| ESALQ_5390 | *M. robertsii* | soil | MH596795 | **43** |
| ESALQ_5389 | *M. robertsii* | soil | MH596798 | **42** |
| ESALQ_5378 | *M. robertsii* | soil | MH596806 | **41** |
| ESALQ_5283 | *M. robertsii* | soil | MH596786 | **40** |
| ESALQ_5179 | *M. anisopliae* | soil | MH596756 | **4** |
| ESALQ_5280 | *M. robertsii* | soil | MH596784 | **38** |
| ESALQ_5285 | *M. robertsii* | soil | MH596787 | **36** |
| ESALQ_5392 | *Metarhizium sp. indet. 1* | soil | MH596824 | **35** |
| ESALQ_5331 | *M. robertsii* | soil | MH596821 | **34** |
| ESALQ_5381 | *M. robertsii* | soil | MH596802 | **33** |
| ESALQ_5375 | *M. robertsii* | soil | MH596809 | **32** |
| ESALQ_5321 | *M. robertsii* | soil | MH596790 | **31** |
| ESALQ_5350 | *M. robertsii* | soil | MH596816 | **30** |
| ESALQ_4992 | *M. robertsii* | soil | MH596766 | **29** |
| ESALQ_5384 | *M. robertsii* | soil | MH596800 | **28** |
| ESALQ_5032 | *M. anisopliae* | soil | MH596737 | **27** |
| ESALQ_5339 | *M. brunneum* | soil | MH596832 | **26** |
| ESALQ_5337 | *M. anisopliae* | soil | MH596736 | **25** |
| ESALQ_5356 | *M. anisopliae* | soil | MH596744 | **24** |
| ESALQ_5334 | *M. anisopliae* | soil | MH596734 | **23** |
| ESALQ_5252 | *M. anisopliae* | soil | MH596760 | **22** |
| ESALQ_5394 | *M. anisopliae* | soil | MH596846 | **21** |
| ESALQ_1668 | *M. anisopliae* | soil | MH596714 | **20** |
| ESALQ_5264 | *M. anisopliae* | soil | MH596762 | **19** |
| ESALQ_5240 | *M. robertsii* | soil | MH596771 | **18** |
| ESALQ_5253 | *M. robertsii* | soil | MH596774 | **17** |
| ESALQ_5379 | *M. robertsii* | soil | MH596807 | **16** |
| ESALQ_1665 | *M. anisopliae* | soil | MH596715 | **15** |
| ESALQ_1658 | *M. robertsii* | soil | MH596718 | **14** |
| ESALQ_5310 | *M. anisopliae* | soil | MH596765 | **130** |
| ESALQ_5258 | *M. anisopliae* | soil | MH596761 | **13** |
| ESALQ_5383 | *M. robertsii* | soil | MH596804 | **12** |
| ESALQ_1652 | *M. robertsii* | soil | MH596717 | **11** |
| ESALQ_4978 | *M. anisopliae* | soil | MH596731 | **109** |
| ESALQ_5245 | *M. anisopliae* | insect | MH596758 | **108** |
| ESALQ_5354 | *M. anisopliae* | insect | MH596743 | **107** |
| ESALQ_5251 | *M. anisopliae* | insect | MH596753 | **104** |
| ESALQ_5362 | *M. anisopliae* | insect | MH596751 | **103** |
| ESALQ_5120 | *M. anisopliae* | insect | MH596749 | **102** |
| ESALQ_5357 | *M. anisopliae* | soil | MH596745 | **101** |
| ESALQ_5351 | *M. anisopliae* | soil | MH596740 | **100** |
| ESALQ_5281 | *M. robertsii* | soil | MH596785 | **10** |
| ESALQ_5382 | *M. robertsii* | soil | MH596803 | **1** |
| ESALQ_1648 | *M. robertsii* | soil | MH596716 |  |
| ESALQ_1657 | *Metarhizium sp. indet. 1* | soil | MH596726 |  |
| ESALQ_1659 | *Metarhizium sp. indet. 1* | soil | MH596727 |  |
| ESALQ_1671 | *M. anisopliae* | soil | MH596712 |  |
| ESALQ_1679 | *M. robertsii* | soil | MH596722 |  |
| ESALQ_1682 | *M. robertsii* | soil | MH596723 |  |
| ESALQ_1685 | *M. robertsii* | soil | MH596725 |  |
| ARSEF_1914 | *M. majus* |  | [KJ398801](https://www.ncbi.nlm.nih.gov/nucleotide/KJ398801.1?report=genbank&log$=nuclalign&blast_rank=1&RID=MBYDEPPP015) |  |
| ARSEF_2107 | *M. brunneum* |  | [EU248855](https://www.ncbi.nlm.nih.gov/nucleotide/EU248855.1?report=genbank&log$=nuclalign&blast_rank=4&RID=MBY95UV701R) |  |
| ARSEF_727 | *M. robertsii* |  | [KX342746](https://www.ncbi.nlm.nih.gov/nucleotide/KX342746.1?report=genbank&log$=nuclalign&blast_rank=13&RID=MBYB70DY01R) |  |
| ARSEF_7488 | *M. lepidiotae* |  | [EU248865](https://www.ncbi.nlm.nih.gov/nucleotide/EU248865.1?report=genbank&log$=nuclalign&blast_rank=1&RID=MBYGMC1Y014) |  |
| CBS_257.90 | *M. pingshaense* |  | [U248850](https://www.ncbi.nlm.nih.gov/nucleotide/EU248850.1?report=genbank&log$=nuclalign&blast_rank=50&RID=MBY5CAPA01R) |  |
| CBS_258.90 | *M. guizhouense* |  | [EU248862](https://www.ncbi.nlm.nih.gov/nucleotide/EU248862.1?report=genbank&log$=nuclalign&blast_rank=3&RID=MBYEXZZC014) |  |
| ESALQ1636 | *Metarhizium sp. indet. 2* | | KP027982 |  |
| ARSEF_7501 | *M. robertsii* |  | [EU248849](https://www.ncbi.nlm.nih.gov/nucleotide/EU248849.1?report=genbank&log$=nuclalign&blast_rank=1&RID=MBYJXKFB014) |  |
| ARSEF_6347 | *M. anisopliae* |  | [EU248881](https://www.ncbi.nlm.nih.gov/nucleotide/EU248881.1?report=genbank&log$=nuclalign&blast_rank=17&RID=MBXYCY02015) |  |
| ARSEF_7487 | *M. anisopliae* |  | [DQ463996](https://www.ncbi.nlm.nih.gov/nucleotide/DQ463996.2?report=genbank&log$=nuclalign&blast_rank=11&RID=MBY223KB01R) |  |
| IP145 | *M. robertsii* |  | JQ061242 |  |
| ESALQ1639 | *M. anisopliae* |  | KP027956 |  |
| ESALQ1614 | *M. anisopliae* |  | [KP027962](https://www.ncbi.nlm.nih.gov/nucleotide/KP027962.1?report=genbank&log$=nuclalign&blast_rank=1&RID=KFMBVVAP014) |  |
| ESALQ_1620 | *M. robertsii* |  | KP027970 |  |
| ESALQ_1621 | *M. robertsii* |  | KP027980 |  |
| ESALQ_1632 | *M. robertsii* |  | KP027976 |  |

**Supplementary Table S4: Number of isolates recovered from soil and root from the sprayed and unsprayed plot by sampling date**

|  | Before fungus application | | | After fungus application | | | | | |
| --- | --- | --- | --- | --- | --- | --- | --- | --- | --- |
|  | **90 days** | | | **30 days** | | | **90 days** | | |
| **plot** | **soil** | **root** | **total** | **soil** | **root** | **total** | **soil** | **root** | **total** |
| **sprayed** | 49 | 6 | 55 | 43 | 15 | 58 | 38 | 0 | 38 |
| **unsprayed** | 64 | 5 | 69 | 47 | 3 | 50 | 61 | 0 | 61 |
| **Total** | 113 | 11 | 124 | 90 | 18 | 108 | 99 | 0 | 99 |
